# Reconstructing the muscular ground pattern of phylactolaemate bryozoans: first data from gelatinous representatives

**DOI:** 10.1186/s12862-017-1068-y

**Published:** 2017-11-07

**Authors:** Natalie Gawin, Andreas Wanninger, Thomas Schwaha

**Affiliations:** Faculty of Life Sciences, Department of Integrative Zoology, University of Vienna, Althanstraße 14, 1090 Vienna, Austria

**Keywords:** Muscle evolution, Freshwater Bryozoa, Lophophorata, Lophophore

## Abstract

**Background:**

Phylactolaemata is commonly regarded the earliest branch within Bryozoa and thus the sister group to the other bryozoan taxa, Cyclostomata and Gymnolaemata. Therefore, the taxon is important for the reconstruction of the bryozoan morphological ground pattern. In this study the myoanatomy of *Pectinatella magnifica*, *Cristatella mucedo* and *Hyalinella punctata* was analysed by means of histology, f-actin staining and confocal laser-scanning microscopy in order to fill gaps in knowledge concerning the myoanatomy of Phylactolaemata.

**Results:**

The retractor muscles and muscles of the aperture, gut, body wall, tentacle sheath, lophophore constitute the most prominent muscular subsets in these species. The lophophore shows longitudinal muscle bands in the tentacles, lophophoral arm muscles, epistome musculature and hitherto undescribed muscles of the ring canal. In general the muscular system of the three species is very similar with differences mainly in the body wall, tentacle sheath and epistome. The body wall contains an orthogonal grid of musculature. The epistome exhibits either a muscular meshwork in the epistomal wall or muscle fibers traversing the epistomal cavity. The whole tentacle sheath possesses a regular mesh of muscles in *Pectinatella* and *Cristatella*, whereas circular muscles are limited to the tentacle sheath base in *Hyalinella*.

**Conclusion:**

This study is the first to describe muscles of the ring canal and contributes to reconstructing muscular features for the last common ancestor of all bryozoans. The data available suggest that two longitudinal muscle bands in the tentacles, as well as retractor muscles and longitudinal and circular muscles in the tentacle sheath, were present in the last common bryozoan ancestor. Comparisons among bryozoans shows that several apomorphies are present in the myoanatomy of each class- level taxon such as the epistomal musculature and musculature of the lophophoral arms in phylactolaemates, annular muscles in cyclostomes and parietal muscles in gymnolaemates.

**Electronic supplementary material:**

The online version of this article (10.1186/s12862-017-1068-y) contains supplementary material, which is available to authorized users.

## Background

**Fig. 1:**
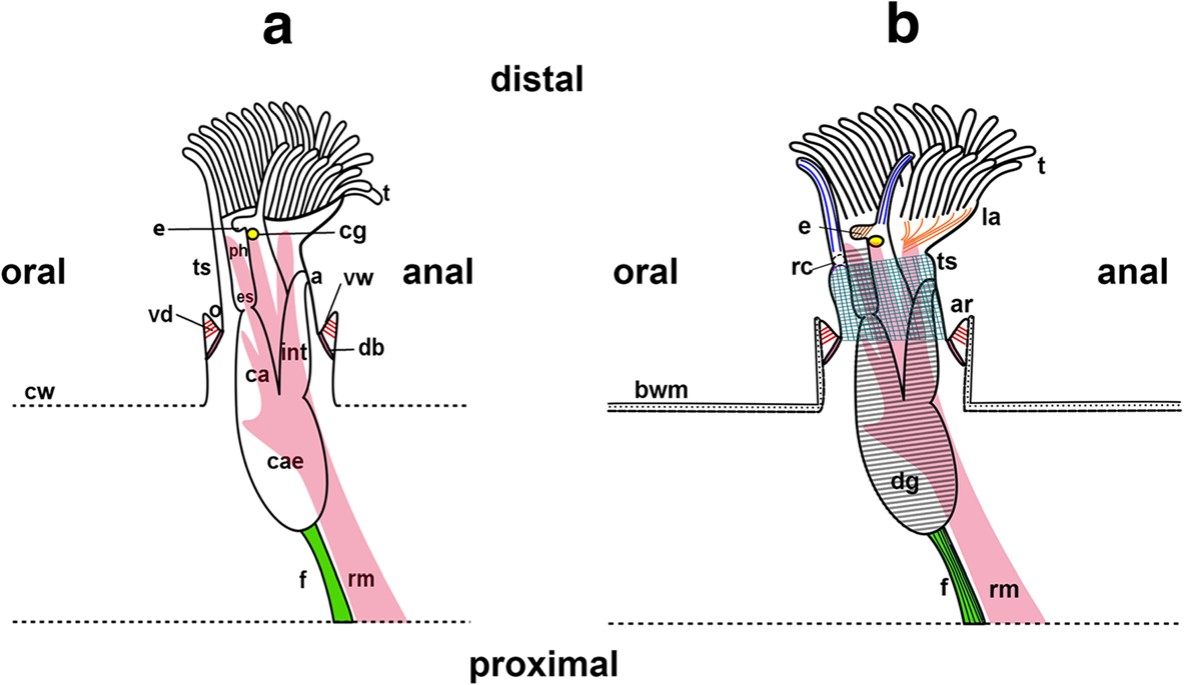
Schematic overview of one everted phylactolaemate zooid with the main structures and musculature shown. **a**: The polypide is surrounded by the protective cystid wall. The lophophore carries all tentacles. The u-shaped digestive tract is divided into pharynx, esophagus, cardia, caecum and intestine, which terminates with the anus in the tentacle sheath outside the lophophore. The paired retractor muscles originate from the proximal body wall and insert at several locations of the polypide. When the animal is retracted the tentacle crown is surrounded by the tentacle sheath. The connection between the outer cystid and the tentacle sheath is formed by the vestibular wall. **b**: Schematic overview of the main parts of the muscular system in *Pectinatella magnifica*. The tentacles contain two longitudinal muscle bands, a frontal (dark blue) and a abfrontal (blue) one. The epistome also possesses distinct musculature (brown). In *Pectinatella* they traverse the coelomic cavity. The lophophoral arms contain longitudinal muscle fibers (orange). In the ring canal muscular elements (purple) are at the base and on the distal side. The digestive tract contains exclusively ring musculature (grey) and the funiculus (green) is supplied by fine longitudinal muscles (black). The body wall contains two or three layers of musculature in *Pectinatella* (black dashed lines). The tentacle sheath contains a fine mesh of longitudinal and circular musculature (cyan). Abbreviations: a - anus, ar – aperture region, bw – body wall, ca - cardia, cae - caecum, cw - cystid wall, db - duplicature bands, dg - digestive tract, e - epistome, es - esophagus, f - funiculus, cg - cerebral ganglion, int - intestine, la – lophophoral arm, o - orifice, ph - pharynx, rc – ring canal, rm. - retractor muscle, t – tentacles, ts - tentacle sheath, vd – vestibular dilatators, vw - vestibular wall

Bryozoa, also called Ectoprocta, includes small, aquatic invertebrates that inhabit both marine and freshwater habitats. Three taxa are commonly recognized: the exclusively freshwater- Phylactolaemata, the marine Stenolaemata, with the only recent group Cyclostomata, and the mostly marine Gymnolaemata [1]. They are sessile, colonial filter feeders that consist of individual zooids. Each zooid is divided into a cystid and a polypide with the latter being retractable into the former – a characteristic defense behaviour of all bryozoans. The cystid (or body wall) consists of two layers, the outer extracellular secreted layer, called the ectocyst, which, in the case members of the Phylactolaemata, is uncalcified, and the endocyst, which represents the cellular epithelial layers below the ectocyst. The polypide is the retractable part of the animal and consists mainly of the lophophore and the digestive tract (Fig. 1.). The lophophore bears all tentacles which proximally join into the lophophoral base surrounding the mouth opening [1]. The gut is u-shaped and divided into several regions. It is possible to distinguish between pharynx, esophagus, cardia, caecum and intestine/rectum. The anus is located near the mouth opening but outside the lophophore and terminates into the tentacle sheath, as typical for all bryozoans [2] (see also Fig. 1 for orientation). Retraction of the polypide into the protective cystid is due to contraction of the retractor muscle. The tentacle crown and the invaginable tentacle sheath are thus pulled in through an opening called the orifice or aperture into the cystid [1].

Several morphological features are characteristic of phylactolaemate bryozoans: a horseshoe-shaped lophophore with a flap-like epistome above the mouth and two lophophoral arms projecting to the anal side [3], a regular orthogonal grid of body wall muscles [4], and statoblasts – dormant stages for dispersal and overwintering e.g. [5].

Traditionally, Bryozoa has been united with Phoronida and Brachiopoda into the Lophophorata because they share some distinct morphological structures such as the food-gathering lophophore [6]. Molecular studies first verified that all three taxa belong to the Lophotrochozoa (e.g. [7, 8]) and are not associated with deuterostomes as once theorized (e.g. [9]). Different scenarios have been, however, proposed for the Lophophorata. In most of them, ‘Lophophorata’ is not recognized as monophyletic (e.g. [10–12]). Phoronida and Brachiopoda were commonly united as ‘Brachiozoa’ whereas Bryozoa had an ambiguous phylogenetic position (e.g. [13]). Then again, two recent studies support a monophyletic status of the Lophophorata [14, 15]. An alternate hypothesis is a close relationship between Bryozoa and Kamptozoa (Entoprocta) [7]. Recent morphological data using immmunocytochemical stainings to analyse nervous systems in these groups also support a monophyly of Lophophorata with the exclusion of Kamptozoa [16–18].

Phylactolaemata has been considered the earliest branching clade within the Bryozoa and sister taxon to Stenolaemata + Gymnolaemata [3]. However, based on morphological features such as lophophore development or body wall musculature, diphyletic Bryozoa was also suggested, with Phylactolaemata forming a clade with Phoronida [19]. Nevertheless, molecular data support: 1) the monophyly of Bryozoa, 2) Phylactolaemata as earliest diverging clade and 3) the sister group relationship of Phylactolaemata to the other two class-level taxa [20, 21].

The myoanatomy of phylactolaemate bryozoans has been investigated in few taxa. Past studies most notably used staining with methylene blue, observations of living material and transmission electron microscopy (e.g. [1, 4, 22, 23]). The most prominent muscles recognized are the retractors on both sides of the digestive tract (Fig. 1). In the lophophore, several muscle types are present including the tentacle musculature, musculature of the lophophoral arms and epistome musculature. As mentioned above, regular orthogonal grid of circular and longitudinal body wall musculature is typical for these animals. The gut possesses prominent ring muscles and the funiculus (a peritoneal cord connecting the proximal end of the gut with the body wall) contains longitudinal muscles. The apertural region contains several sets of muscles: muscles of the vestibular wall with a sphincter, the vestibulum dilatators and duplicature bands [24–26].

Several studies on the nervous system (e.g. [17, 27, 28]) and the myoanatomy of adult phylactolaemates (e.g. [25]), gymnolaemate ctenostomes [25] and bryozoan larvae (e.g. [29, 30]) show that highly specific histochemical (phallodin) and immunohistochemical (antibody) staining and confocal microscopy are appropriate methods for reconstructing the neuro-muscular system of these animals. Up to date, the myoanatomy of species belonging to 2 of the 6 family-level taxa of Phylactolaemata have been described with confocal microscopy [26]. The investigated families Fredericellidae and Plumatellidae are considered later-branching and whose members (with few exceptions) possess sand- incrusting or chitinous ectocyst walls and also a branching colony type [31]. The remaining species of the other families (Cristatellidae, Pectinatellidae, Lophopodidae and Stephanellidae) have gelatinous ectocysts and mostly more globular colonies than branching ones.

The present study thus extends the information on the myoanatomy within the Phylactolaemata by analysing three gelatinous species belonging to three different families (Cristatellidae, Pectinatellidae, and one of the few gelatinous species of Plumatellidae) by means of f- actin (muscle) staining, confocal microscopy and histology to gain more comparable data concerning the muscular system and to clarify the muscular ground pattern of Phylactolaemata.

## Methods

### Animals

Colonies of *Pectinatella magnifica* were collected in Třeboň area in the Staňkov pond (Czech Republic) and colonies of *Cristatella mucedo* in the New Danube (Vienna) and the Laxenburger pond (Lower Austria). *Hyalinella punctata* was collected in the Laxenburger pond. Young colonies of *Pectinatella* were available after germination of statoblasts under laboratory conditions. Colony parts and zooids were documented with a Nikon SMZ 1500 stereomicroscope (Nikon, Chiyoda, Tokio, Japan). Specimens were anesthetized with 3% magnesium chloride (MgCl_2_). For confocal microscopy, samples were fixed in 4% paraformaldehyde in 0.1 M phosphate buffer (PB) and for light microscopy and histology in 2% glutaraldehyde in 0.01 M phosphate buffer (PB) see also [4]. The animals were stored in PB with 0.01% NaN_3_ at 4 °C until further investigations.

### Immunocytochemical staining and confocal microscopy.

Prior to staining, single zooids were separated from the colony. Due to their large size they were dissected into smaller pieces to increase tissue permeability. Vibratome sections of 100–200 μm thickness from colonies and freshly germinated *Pectinatella* were carried out with a Leica VT 1200 S vibratome (Leica Microsystems, Wetzlar, Germany). Subsequently, specimens were permeabilized by overnight treatment with PB containing 10% Triton-X 100 (PBT). This was followed by f-actin staining with Alexa Fluor 488 phalloidin (Molecular Probes, Eugene, OR) in a dilution of 1:60 and by nuclei staining with 4′,6-diamidino-2-phenylindole (DAPI) (Invitrogen, Carlsbad, CA, USA) in a dilution of 1:120. Specimens were incubated overnight and subsequently 4 washing steps were carried out for 30 min each. Specimens were mounted with Fluoromount G (Southern Biotech, Birmingham, AL, USA) on standard microscope slides.

The samples were scanned with a Leica TCS SP5 II confocal laserscanning microscope (Leica Microsystems, Wetzlar, Germany). The chosen z-step size was 0.5 - 1 μm. FIJI software [32] was used to analyse image stacks, produce maximum intensity projections and for image editing. Additionally, image stacks were analysed using Amira 5.5 (FEI, Oregon, USA) which was also used to produce volume renderings.

### Histology

Specimens were fixed in glutaraldehyde and afterwards treated with 1% osmium tetroxide in distilled water for 1 h. Dehydration followed after several washing steps with distilled water, with acidified 2,2-dimethoxypropane (DMP). Then, three washes with acetone for 15 min each followed. Specimens were embedded in Agar Low viscosity resin with acetone as intermediate (Agar Scientific, Stansted, Essex, UK). Polymerisation followed at 65° for 12 h. Ribbons of semi-thin sections (1 μm thickness) were produced with a Leica UC6 ultramicrotome (Leica Microsystems, Wetzlar, Germany) using a Diatome histo jumbo diamond knife (Diatome, Biel, Switzerland). Afterwards, the sections were stained with 0.1% toluidine blue for 10 s at 65 °C and rinsed with distilled water. The semi-thin sections were analysed with a Nikon E800 light microscope (Nikon, Chiyoda, Tokio, Japan) and photographed with a Nikon Fi2-U3 microscope camera (Nikon, Chiyoda, Tokio, Japan).

## Results

The description of the muscular system is valid for all species as well as for freshly germinated *Pectinatella magnifica* colonies, differences are mentioned where present. In general, the muscular system is very similar and differences are largely present in the body wall and epistome.

### General morphology, body wall musculature, tentacle sheath musculature and retractor muscles

**Fig. 2:**
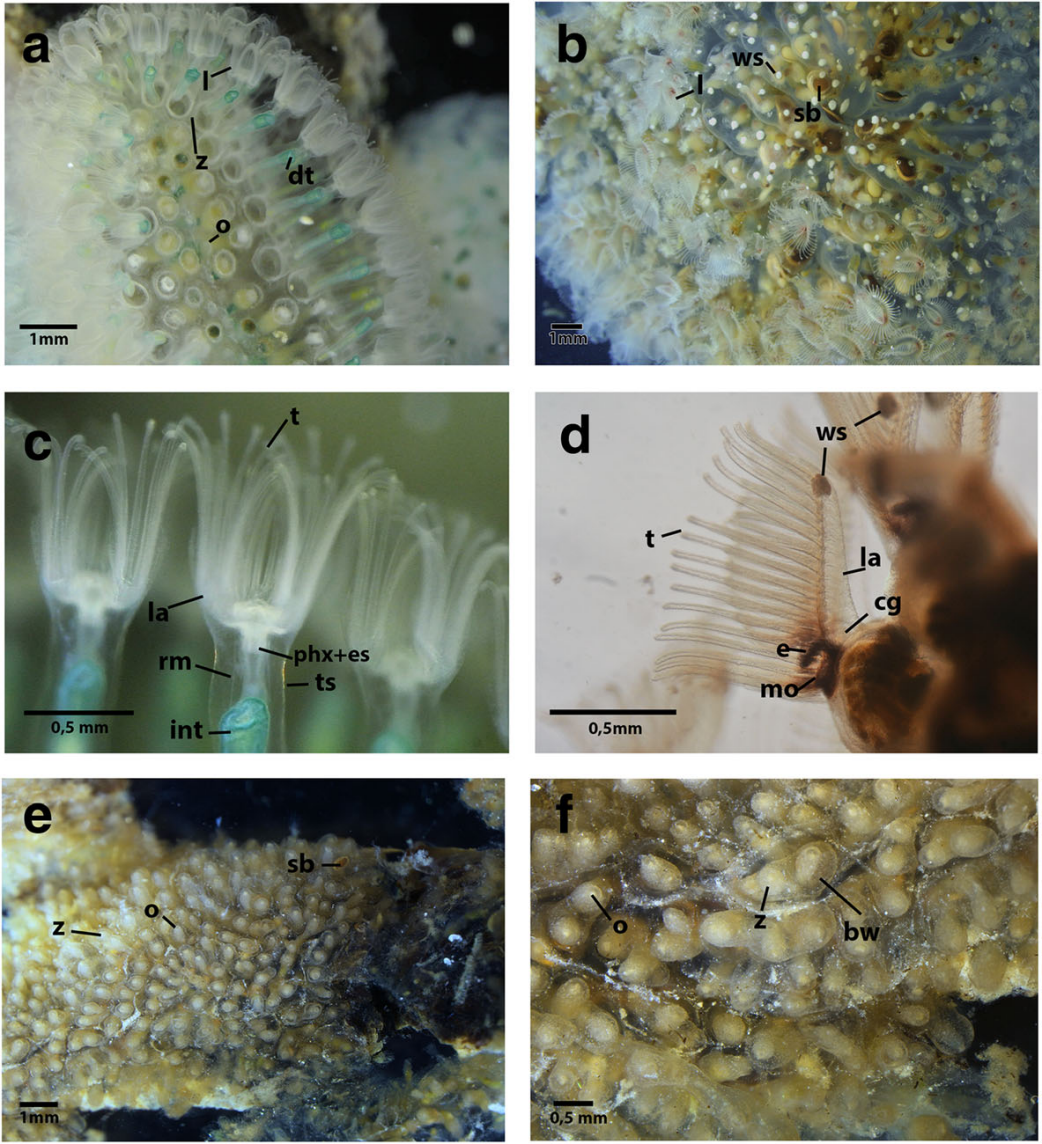
Colony parts and zooids of *Pectinatella magnifica*, *Cristatella mucedo* and *Hyalinella punctata* **a**: Part of the worm-shaped colony of *C. mucedo,* where protruded zooids are visible. The green structures are the digestive tracts of the animals and the prominent lophophore is visible. **b**: Part of a *P. magnifica* colony with protruded zooids. Statoblasts are visible within the colony. White spots are located over the whole colony in a regular pattern. **c**: Detail of *C. mucedo* zooids viewed from the anal side. The lophophoral arms with the tentacles, the tentacle sheath and the retractors are visible. Furthermore, the intestine is visible in green. **d**: Single zooid of *P. magnifica* seen from the lateral side. The area of the epistome and foregut appears heavily pigmented. At the distal tip of the prominent lophophoral arms white spots are visible. **e**: Part of a *H. punctata* colony with retracted zooids. Despite the tight arrangement the branching colony pattern is visible. **f**: Close-up of a colony of *H. punctata* with retracted zooids. The orifice and the gelatinous body wall are visible. Abbreviations: bw – body wall, cg – cerebral ganglion, e- epistome, dt - digestive tract, es – esophagus, int – intestine, l - lophophore, la- lophophoral arms, mo – mouth opening, o - orifice, phx – pharynx, rm. – retractor muscle, sb – statoblast, t - tentacles, ts – tentacle sheath, ws – white spot, z – zooid

Species of *Pectinatella* and *Cristatella* have a very prominent lophophore with large lophophoral arms and the gelatinous ectocyst only on their basal side (Figs. 2c, d). The entire colony of *Cristatella mucedo* is worm-shaped (Fig. 2a). On the colony surface of *P. magnifica* is a regular pattern of white spots, which are glandular complexes (Fig. 2b). In *P. magnifica* each zooid has a typical dark red color around the area of the epistome and foregut (Fig. 2d). *Hyalinella punctata* has a braching-type of colony and the ectocyst is also transparent and gelatinous but it is situated over the whole colony and not only at the basal side (Figs. 2e, f).

**Fig. 3:**
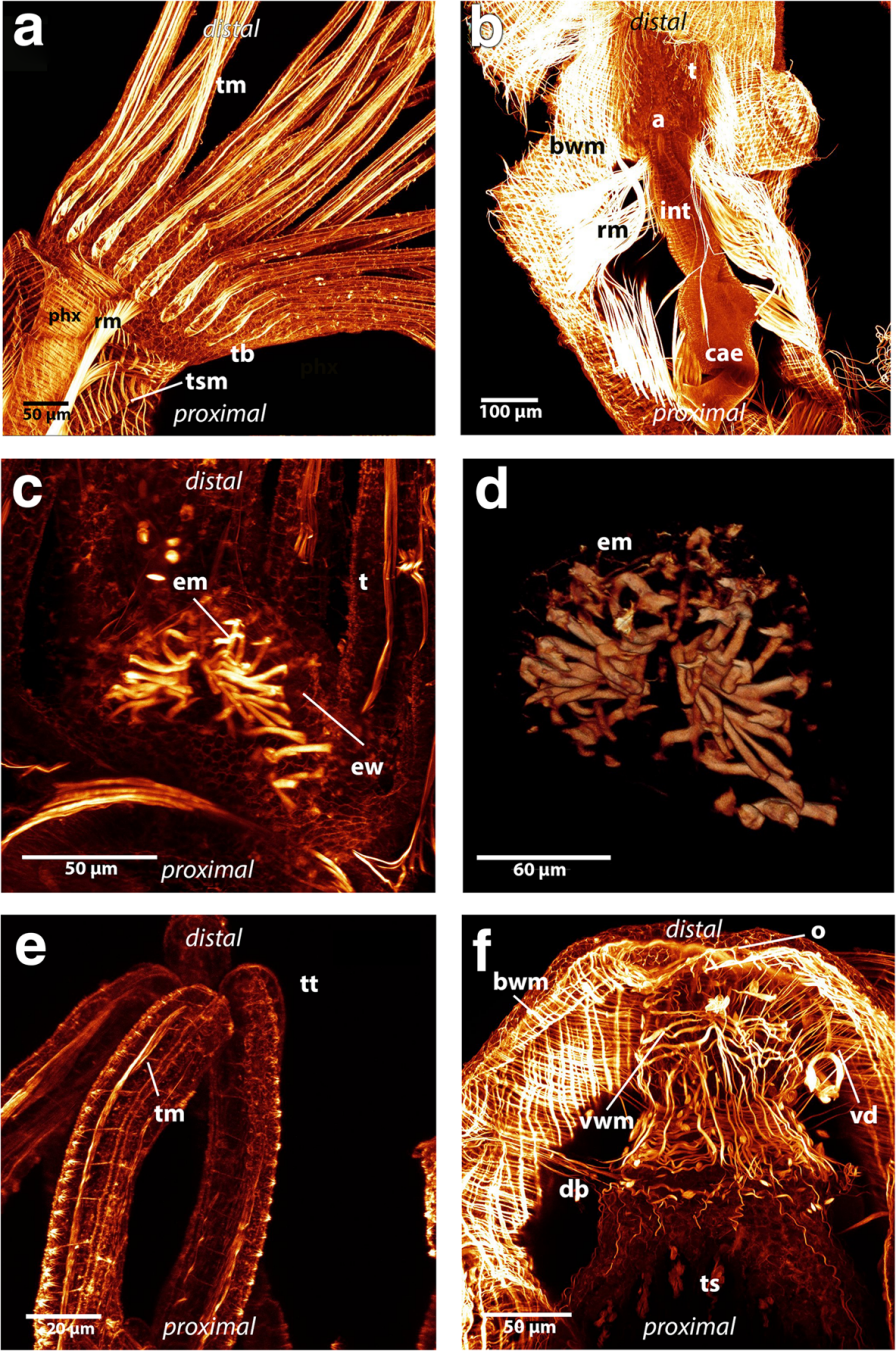
Maximum intensity projections and volume rendering of f-actin staining scans of *Hyalinella punctata*. **a**: Lateral view of the lophophore and part of the tentacle sheath. *Hyalinella* possesses exclusively longitudinal musculature on the distal side of the tentacle sheath. Attachment sites of the retractor are visible at the lophophore and tentacle sheath. Also the abfrontal tentacle muscle band and the circular muscles of the pharynx is shown. **b**: Zooid viewed from the anal side surrounded by retractor muscles and body wall musculature. The retractors are attached to the animal at the tentacle sheath, ganglion, lophophore and at several spots at the digestive tract: pharynx, esophagus, cardia and caecum. The body wall possesses two muscular layers. **c**: The epistome musculature of *Hyalinella* from the proximal side. The muscle fibers are orientated laterally and are situated in the epistomal wall or traverse the coelomic cavity. **d**: Volume rendering of epistomal musculature in *Hyalinella* viewed from the proximal side*.* **e**: Distal ends of tentacles where strong f- actin positive signal in form of “knobs” in the epidermal layer of the tentacles is visible. **f**: Aperture musculature and the circular muscles located at the basal part of the tentacle sheath in *H. punctata*. The vestibular wall contains prominent circular as well as longitudinal musculature. The duplicature bands are attached to the tentacle sheath and body wall. The vestibulum dilatators traverse the coelomic cavity between vestibular wall and body wall. Abbreviations: a - anus, bwm - bodywall musculature, cae – caecum, db – duplicature bands, em - epistome musculature, ew – epistomal wall, int – intestine, o - orifice, phx - pharynx, rm. - retractor muscles, t – tentacle, tb – tentacle base, tm - tentacle musculature, ts – tentacle sheath, tsm - tentacle sheath musculature, tt – tentacle tips, vd – vestibulum dilatators, vwm – vestibular wall musculature

The polypide contains all main organs and consists most notably of the lophophore (the tentacle crown) and the digestive tract. On each side of the digestive tract is a prominent retractor muscle consisting of longitudinal smooth muscles arranged in bundles. The retractor muscles are attached to the polypide at several locations: the lophophoral base, the tentacle sheath, the peritoneum surrounding the ganglion and the oral side of the digestive tract. The retractors are also attached to the pharynx, esophagus, cardia and caecum. They extend along the digestive tract and insert at the basal part of the body wall of the colony (Figs. 1 and 3 b).

**Fig. 4:**
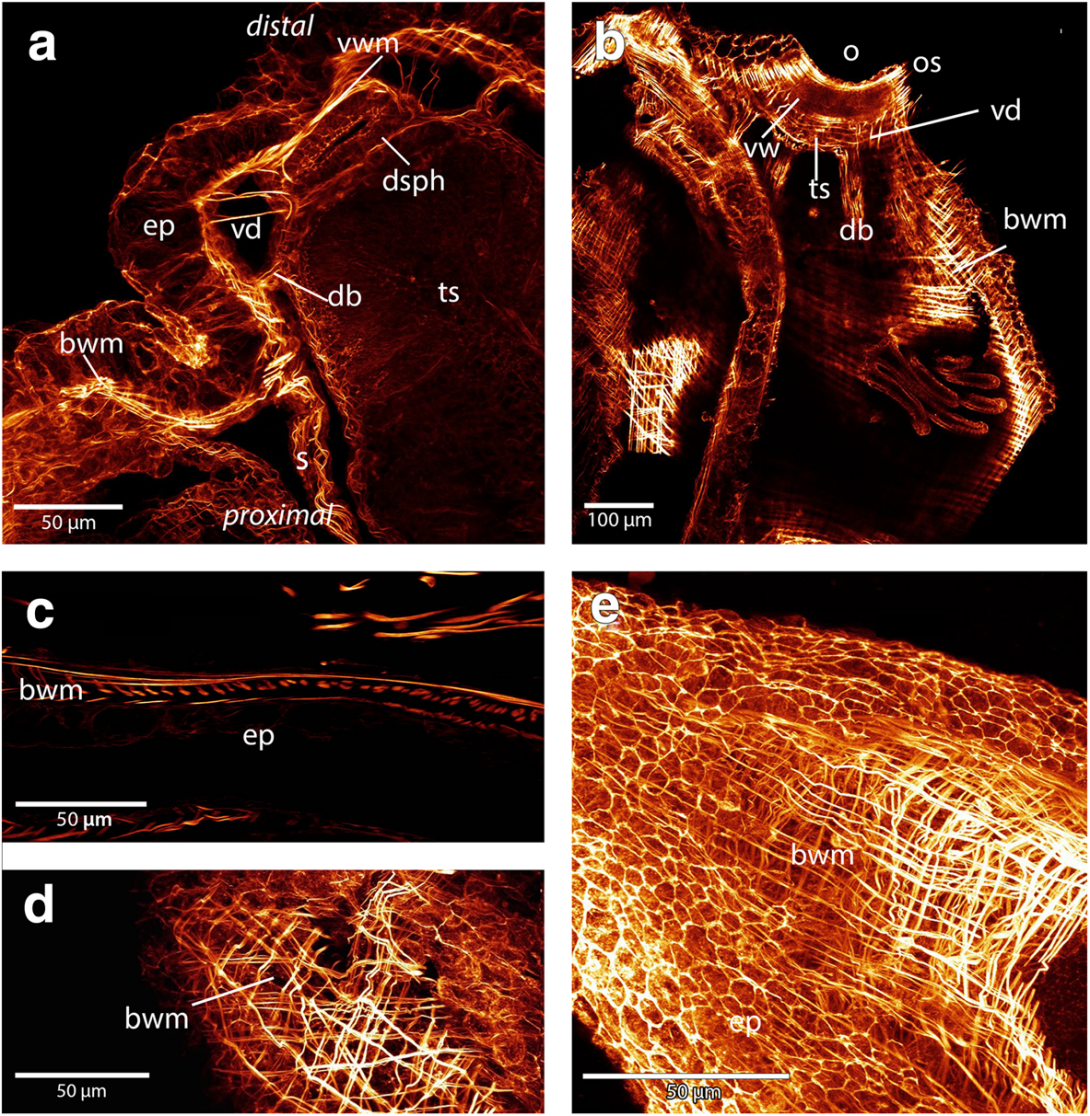
Maximum intensity projections and volume renderings of f-actin stainings of the apertural region and body wall in *Cristatella* and *Pectinatella*. **a**: The musculature of the aperture region in *Cristatella*. The apertural musculature consists of several muscle sets: the duplicature bands which are located between the body wall and tentacle sheath and vestibulum dilatators which are located between the body wall and vestibular wall, which also contains musculature. Between the vestibular wall and tentacle sheath the diaphragm sphincter is situated. **b**: Apertural region in *Pectinatella.* Note that parts of the body wall musculature are removed. The circular arrangement of the duplicature bands and vestibulum dilatators are visible around the vestibular wall and tentacle sheath. **c** and **d**: In *Pectinatella magnifica* a third muscular layer in the body wall exists in some areas. This muscle layer is arranged diagonally to the other two. **e**: Two layers of body wall musculature form the regular muscular mesh in *Cristatella mucedo.* Abbreviations: bwm – body wall musculature, db - duplicature bands, dsph -diaphragmatic sphincter, ep - epidermis, o - orifice, os - orifice sphincter, s – septa, ts - tentacle sheath, vd - vestibulum dilatators, vw - vestibular wall, vwm - vestibular wall musculature

**Fig. 5:**
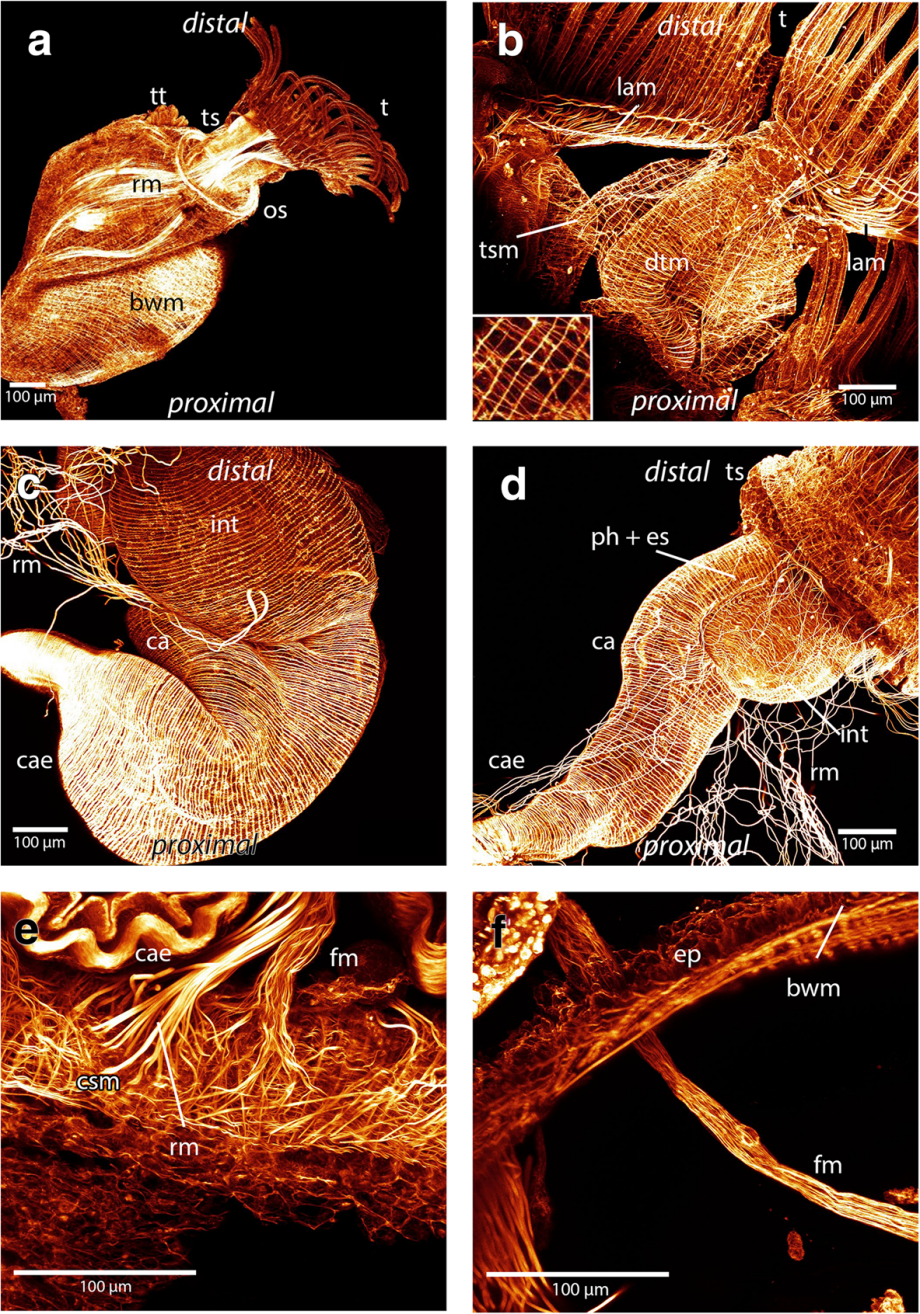
Overview of the musculature in the digestive tract and the funiculus shown in maximum intensity projections and volume renderings of f- actin staining in *Cristatella* and *Pectinatella.* **a**: Overview of the muscular system in a young *Pectinatella* colony shortly after germinating. Two zooids are visible, one is completely protruded. The tentacle crown bears all tentacles. Prominent retractor muscles are visible and the aggregation of ring musculature at the orifice forms the orifice sphincter. The body wall contains prominent musculature. **b**: Oral view of *Cristatella* and lateral view on a lophophoral arm where the intertentacular membrane is visible. Lophophoral arm musculature branches off to the tentacles. The tentacle sheath contains longitudinal and circular musculature, note the detail in the lower left corner of the tentacle sheath musculature. The u-shaped digestive tract contains circular musculature. **c**: Lateral view of the digestive tract of *Pectinatella*. Denser aggregation of the musculature at the caecum is visible. **d**: Lateral view of the digestive tract in *Cristatella*. At the beginning of the digestive tract, at the proximal end of the caecum and at the rectum a denser aggregation of musculature is visible. **e**: Detail of funiculus musculature and retractor muscles in *Cristatella*. Both contain smooth muscle fibers and insert at the basal part of the body wall, in case of *Cristatella* at the creeping sole. **f**: Detail of the funiculus in *Pectinatella* where the fine longitudinal muscles are visible. Abbreviations: bwm – body wall musculature, ca - cardia, cae - caecum, csm - creeping sole musculature, dtm - digestive tract musculature, es - esophagus, ep - epidermis, fm - funiculus musculature, int - intestine, lam - lophophoral arms musculature, os - orifice sphincter, ph - pharynx, rm. - retractor muscle, t - tentacles, ts -tentacle sheath, tsm - tentacle sheath musculature, tt - tentacle tips

The body wall musculature consists of two layers, an outer circular layer and an inner longitudinal layer. These two layers form a regular grid (Figs. 3b, f and 4e). In some areas of the body wall of *P. magnifica* a third layer of diagonally arranged muscles is situated underneath the regular grid of circular and longitudinal musculature (Figs. 4c and d). This layer is present in different locations from the area around the orifice to the basal side but a regular pattern could not be observed. In *C. mucedo* the musculature of the body wall on the proximal side, where the creeping sole is directed towards the substrate, possesses a denser grid of muscles than on the distal side (not touching the substrate) (Fig. 5 e). In the densely aggregated zooids of the worm-shaped colony of *C. mucedo*, septa that partially separate zooids within the colonies also possess the same layers of musculature as observed in the body wall. These septa are situated laterally in each zooid (Fig. 4 a). The other two analysed species lack these septa.

The tentacle sheath surrounds the tentacle crown when the animal is retracted. In protruded zooids it attaches distally to the lophophoral base and proximally into the vestibular wall (Figs. 1 and 3a). The tentacle sheath of *P. magnifica* and *C. mucedo* possesses longitudinal and circular muscles that form a fine regular mesh. The longitudinal fibers are more prominent than the circular fibres (Figs. 1b and 5b). In *H. punctata* the circular muscle fibers are restricted to the base of the tentacle sheath and the rest of the tentacle sheath only possesses longitudinal musculature (Fig. 3a, f).

### Aperture musculature

The orifice is situated on the distal side of the body wall (Fig. 1). Around the orifice a dense aggregation of circular muscles that forms the orifice sphincter (Figs. 4b and 5a). The vestibular wall is an invagination of the body wall that forms a cavity, the so-called vestibule. The musculature of the vestibular wall is formed by more prominent longitudinal muscles but also by circular muscles in *P. magnifica* and *C. mucedo*, whereas in *H. punctata* circular muscles appear more prominent than longitudinal muscles (Fig. 3f). When the animal is retracted, the vestibular wall continues in the proximal direction into the tentacle sheath that surrounds the tentacle crown. The diaphragmatic sphincter is positioned between the two latter structures. It is formed by circular muscles (Fig. 4a). The vestibulum dilatators are single, smooth, longitudinal muscles that are arranged radially around the entire vestibular wall and traverse the coelomic cavity between the vestibular wall and the body wall. The duplicature bands also traverse the coelomic cavity and are located proximally of the vestibulum dilatators. In contrast to the vestibular dilatators, these are peritoneal bands supplied with smooth longitudinal muscles. They extend from the tentacle sheath towards the body wall (Figs.1, 4 a, b and 3f).

### Musculature of the digestive tract and funiculus

The digestive tract is u-shaped and begins with a mouth that is situated at the lophophoral base. The regions of the gut are the pharynx, esophagus, cardia, caecum and intestine (Fig. 1). The funiculus is a peritoneal cord that originates at the proximal tip of the caecum (Figs. 1 and 5e, f). This tubular peritoneal strand contains several smooth longitudinal muscles that extend from the proximal caecum to the body wall. In *C. mucedo* it is always situated above the creeping sole (Figs. 1 and 5e, f). The musculature of the digestive tract is exclusively circular (Figs. 1b and 5c, d). Areas of denser aggregation of the circular muscles can be found in the pharynx and at the proximal end of the caecum. The cardia and intestine have more loosely arranged muscles. The anus is also supplied with circular muscles. In the pharynx and caecum the muscles appear to be striated. The intestine is supplied by smooth musculature and esophagus and cardia contain smooth or striated musculature (Fig.5c, d).

### Musculature of the lophophore

**Fig. 6:**
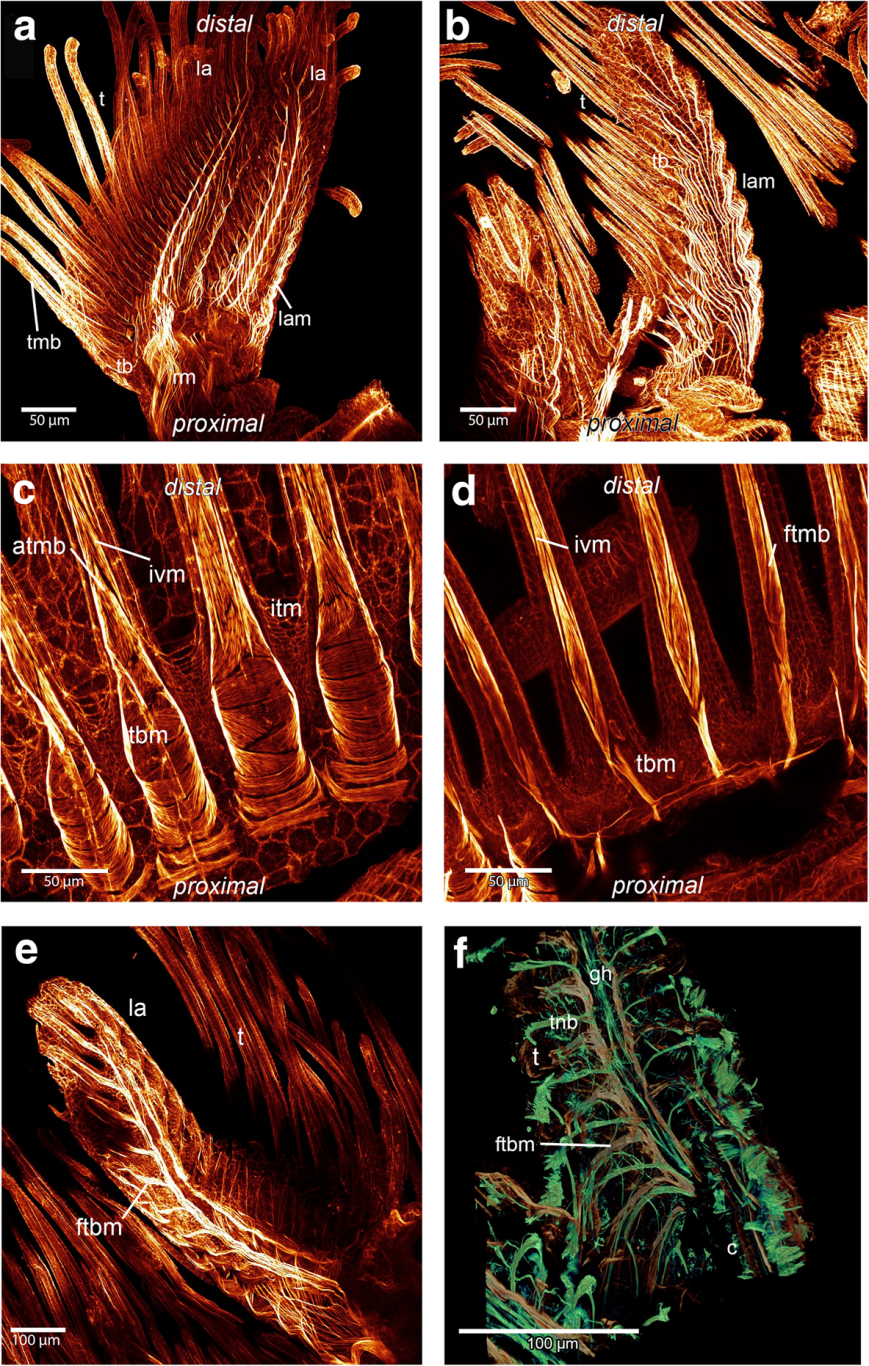
Maximum intensity projections and volume renderings of f-actin staining and alpha-tubulin staining of the lophophoral base and tentacles in *Cristatella* and *Pectinatella*. **a**: Overview of the lophophore from the proximal side of *Cristatella.* The lophophoral arm musculature is visible and runs to the distal tip of the lophophoral arms. These fibers supply the lateral tentacles which are situated at the margin of the lophophoral arms. Part of the retractor muscle is visible. From the retractor muscles two muscle bundles originate which run along the lophophoral base and branch off at the rootlets of the frontal muscle bands. **b**: View of a lophophoral arm of *Pectinatella* from the proximal side where the longitudinal musculature of the lophophoral arms is visible. These muscles branch off to each tentacle base. **c**: Detail of the tentacle base from the abfrontal side in *Pectinatella*. Each abfrontal muscle starts with diagonally arranged musculature at the base of the tentacle and more distally is made of inverted “v” muscles. **d**: Detail of the frontal muscle bands in the tentacle of *P. magnifica* which is also composed of inverted “v” muscles. **e**: One lophohopral arm of *P. magnifica* from the distal view. The rootlets of the lateral tentacles branch off from two main muscle bundles that run along the lophophoral arms. **f**: Detail of one lophophoral arm in *C. mucedo*. The nervous system and cilia of the tentacles are in green and the musculature in reddish-brown. Medially of the lophophoral arm the ganglionic horn is situated and on both sides two muscle bundles from which the frontal muscle base of the tentacle branches off are visible. Abbreviations: atmb - abfrontal tentacle muscle band, c - cilia, ftbm - frontal tentacle base muscle, ftmb - frontal tentacle muscle band, gh - ganglionic horn, itm - intertentacular membrane, ivm - inverted “v” muscle, la - lophophoral arm, lam - lophophoral arm musculature, rm. – retractor muscles, t - tentacle, tb - tentacle base, tbm - tentacle base musculature, tnb - tentacle neurite bundles

**Fig. 7:**
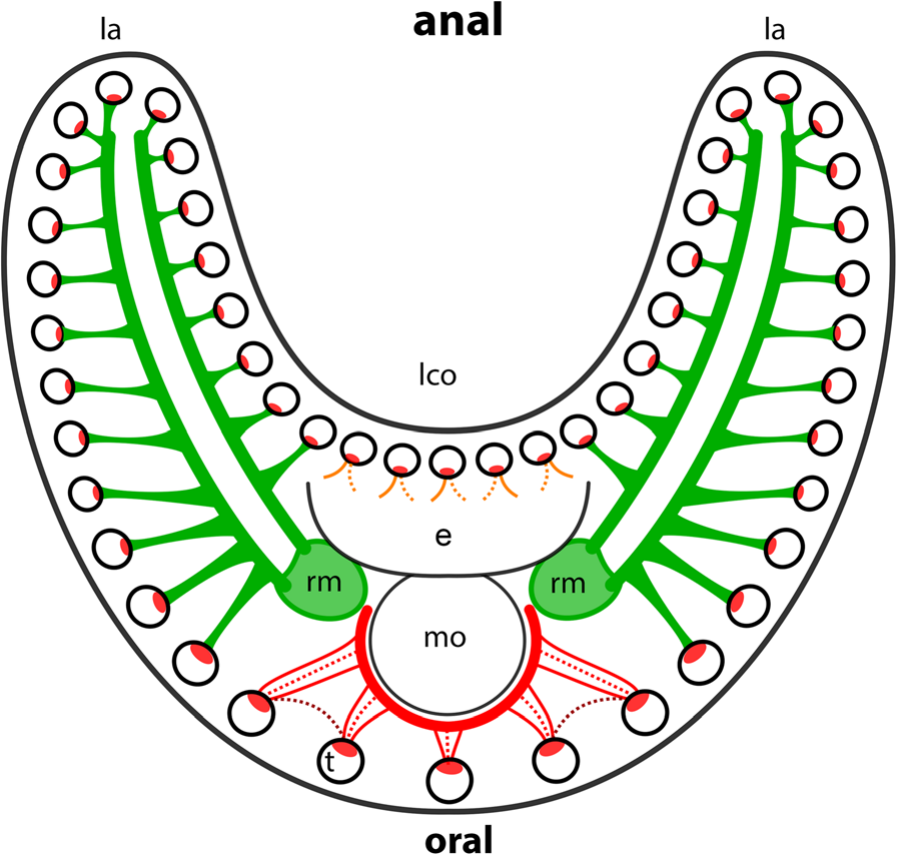
Schematic overview of the frontal tentacle musculature in *Cristatella mucedo* and *Pectinatella magnifica* viewed from distal. Red: inside the tentacle the frontal muscle band is visible. At the frontal, oral tentacles two or three muscular rootlets are present at the base. The latter are connected with a muscle surrounding the pharynx. Cross-connections could be identified in *Cristatella* (dashed lines, dark red). Orange: One or two bases of the tentacles are situated above the epistome and face the lophophoral concavity. Green: two muscle fiber bundles extend as part of the retractor muscle (light green) and run along the lophophoral arms. From this two muscle fiber bundles smaller ones branch off to each lateral tentacle and form the frontal bases. Abbreviations: e - epistome, la - lophophoral arms, lco - lophophoral concavity, mo - mouth opening, rm. - retractor muscle, t - tentacles

In the lophophore, four sets of muscles are present in all three species: musculature of the lophophoral arms, tentacles, epistome and ring canal (Figs. 1b, 6 and 7).

The muscles of the lophophoral arms are formed by longitudinal muscles. In *P. magnifica* and *C. mucedo* they reach into the distal tip of the lophophoral arms and are very prominent. Several muscles are orientated on each lateral side of the lophophoral arm and branch off to each tentacle base on the abfrontal side. In some stainings this musculature appears cross-striated (Figs. 1b and 6a, b). In *H. punctata,* the musculature of the lophophoral arms is poorly developed and consists of delicate longitudinal muscles positioned at the base of the lophophoral arms (Additional 1: Figure S1 c, d).

The musculature of the tentacles is formed by the frontal and the abfrontal longitudinal muscle bands (Figs. 1b, 3a and 6c, d). The muscles of the tentacles are associated with the peritoneal layer that surrounds the coelomic cavity of the tentacles. The frontal muscle bands face the mouth opening and the abfrontal muscles are present on the opposite side. Alongside the tentacles, the two muscle bands are supplied by stacked muscles in the form of an inverted ‘v’ and terminate in the tip of the tentacle. The whole musculature of the tentacle appears striated in *C. mucedo* and *P. magnifica*. Between the proximal area of the tentacles is the intertentacular membrane (Fig. 6c, d). In *H. punctata,* rosette-shaped structures rich in f-actin are present on the surface of the epidermal layer in the tentacles (Fig. 3e).

The abfrontal tentacle base is supplied by several muscles of the lophophoral arm musculature that has an identical appearance over the whole length of the lophophore in *C. mucedo* and *P. magnifica* (Fig. 6 b). In *H. punctata* there is no connection between the bases of the abfrontal tentacle muscles and the musculature of the lophophoral arms. Obliquely arranged muscles are situated at the base of each tentacle on the abfrontal side in all three species (Figs. 3a and 6b, c). Only the abfrontal muscles of the tentacles which face the lophophoral concavity, which is the space limited by the inner margin of the two lophophoral arms, is associated with the epistomal musculature (Fig, 8a, b). The frontal tentacle muscles differ in the oral tentacles, the lateral tentacles and the tentacles which face the lophophoral concavity (Figs. 6 and 7). The frontal muscle band originates at the base of the tentacle and emanates more distally than the abfrontal muscle band (Fig. 6c, d). The oral tentacles contain two or three rootlets in *C. mucedo* and *P. magnifica* and always two in *H. punctata* which do not originate directly from the pharyngeal epithelium but from a muscle that surrounds the pharynx on the oral side (Additional file 1: Figure S1 a). In *C. mucedo,* cross-connections were occasionally located between the rootlets of the oral tentacles but not in a consistent pattern (Fig. 7). Stainings of the tentacles in the lophophoral concavity above the epistome were always very weak but one or two rootlets at the frontal muscles could be recognized in *P. magnifica* and *C. mucedo* and always two in *H. punctata*. In general the tentacle musculature in this area emerges from the musculature of the epistome.

**Fig. 8:**
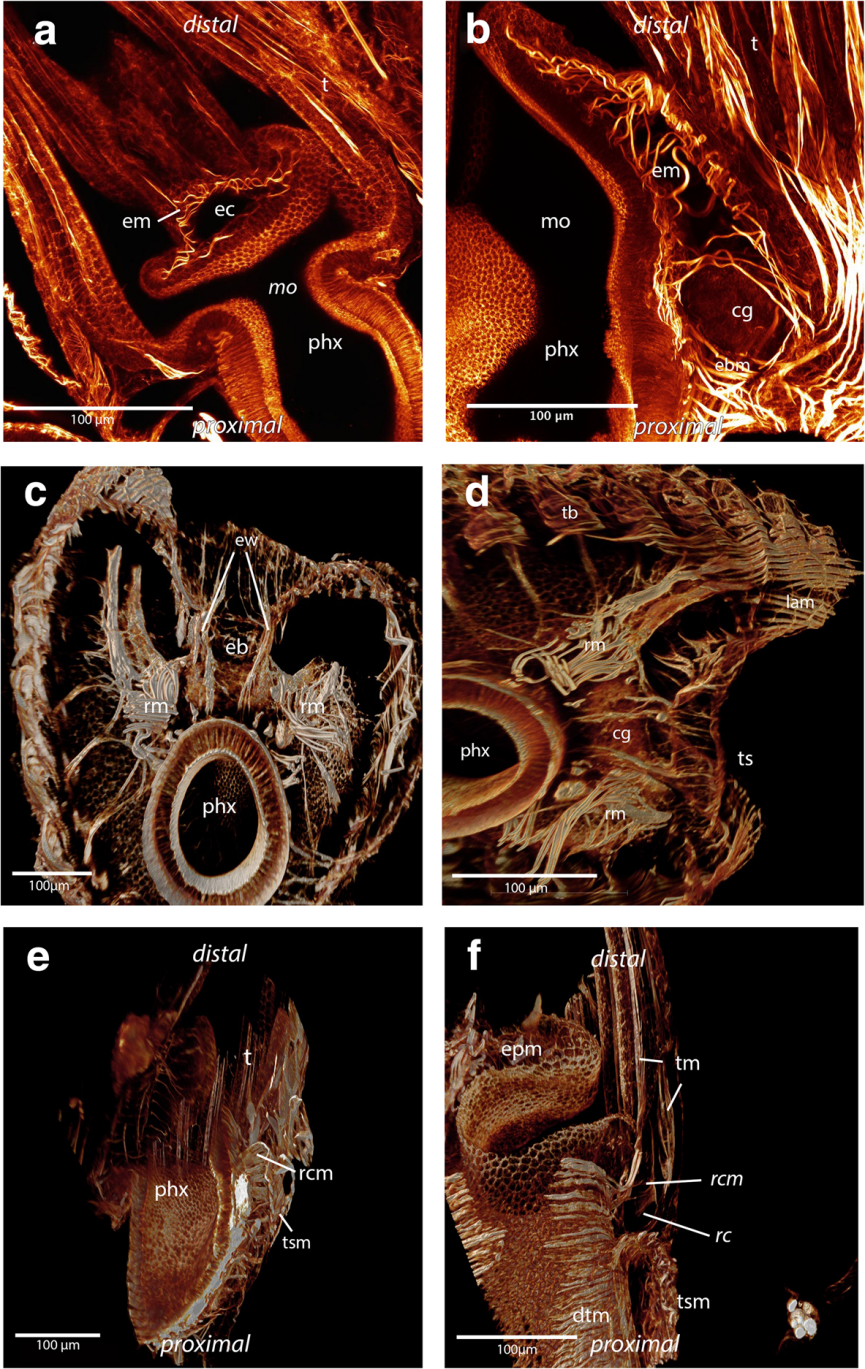
Maximum intensity projections and volume renderings of f actin staining of the epistome and ring canal in *Cristatella mucedo* and *Pectinatella magnifica*. **a**: Epistomal musculature of *Cristatella mucedo* forms a basket within the coelomic cavity in the epistome. **b**: Epistomal musculature of *Pectinatella magnifica* traverses the cavity of the epistome with additional fine fibers in the epistomal wall visible. In the epistomal wall muscle fibers originate at the pharynx and ganglion. **c**: Part of the lophophoral base of *C. mucedo* where the connection of the epistome with the trunk coelom is visible. At the base of the epistome smooth muscle fibers run between the pharyngeal epithelium and tentacle sheath in the wall of the epistomal canal. **d**: View of the lophophoral base in *C. mucedo* from where muscle fibers originate from the pharyngeal epithelium and ganglion and insert at the tentacle sheath. **e**: Ring canal musculature in *C. mucedo*. It consists of muscle fibres which originate from the pharyngeal epithelium and run into the tentacle sheath and very thin muscles in an intertentacular position. **f**: Ring canal musculature in *C. mucedo* consists of more prominent muscle fibers at the base of the canal which originate from the pharyngeal epithelium and pass into the tentacle sheath as well as thin fibres at the distal side of the canal with an intertentacular position. Abbreviations: cg - cerebral ganglion, dtm - digestive tract musculature, eb - epistomal base, ec - epistomal cavity, epm - epistome base musculature, em - epistome musculature, ew – epistomal wall, mo - mouth opening, phx - pharynx, rm. - retractor muscles, rc - ring canal, rcm - ring canal musculature, t - tentacle, tb – tentacle base, tm - tentacle musculature, ts – tentacle sheath, tsm - tentacle sheath musculature

In *C. mucedo* and *P. magnifica* two bundles of smooth muscles extend medially into the lophophoral arms and are situated next to the ganglionic horns (Figs. 6e, f and 7) where smaller bundles branch off and form the rootlets of the frontal muscles of the lateral tentacles. These two prominent bundles of muscles are formed by extensions of the retractor muscles that insert near the epistome (Figs. 7 and 8c, d). These extensions are missing in *H. punctata* and the lateral tentacles possess one rootlet at the frontal position.

The epistome above the mouth opening of *Cristatella mucedo* is flatter than the epistome of *Pectinatella magnifica.* The musculature is formed by several smooth muscles in the shape of a muscular basket (Figs. 1b and 8a). In contrast to *C. mucedo, P. magnifica* possesses smooth muscles that traverse the epistomal coelomic cavity. Furthermore, some fine muscles are situated in the epistomal wall (Fig. 8b). At the base of the epistome in the epistomal wall smooth muscles are situated which originate from the pharyngeal epithelium and the cerebral ganglion (brain) and terminate at the tentacle sheath (Fig. 8c, d). In *H. punctata* the musculature of the epistome is formed by a mixture of muscles which are located in the epistomal wall (like in *C. mucedo*) and fibers which are traversing the coelomic cavity of the epistome (like in *P. magnifica*). The fibers are orientated laterally (Fig. 3c, d).

The ring canal is located at the base of the oral tentacles and is separated from the body coelom similarly to the forked canal, which is situated above the epistome at the base of the lophophoral concavity (cf. [24]). Muscles are present on the proximal side of the ring canal. These muscles originate from the pharyngeal epithelium, extend through the proximal peritoneal lining of the ring canal and pass into the tentacle sheath. In *C. mucedo* and *P. magnifica* thin muscular elements with weak signal are also distinguishable on the distal side of the canal in an intertentacular position (Fig. 8e, f). In *Hyalinella* some variability in the ring canal musculature could be observed. Single muscles traverse the ring canal medially (Additional file 1: Fig. S1 b), but only in a few specimens and always without a regular pattern.

### White spots and vestibular pore

**Fig. 9:**
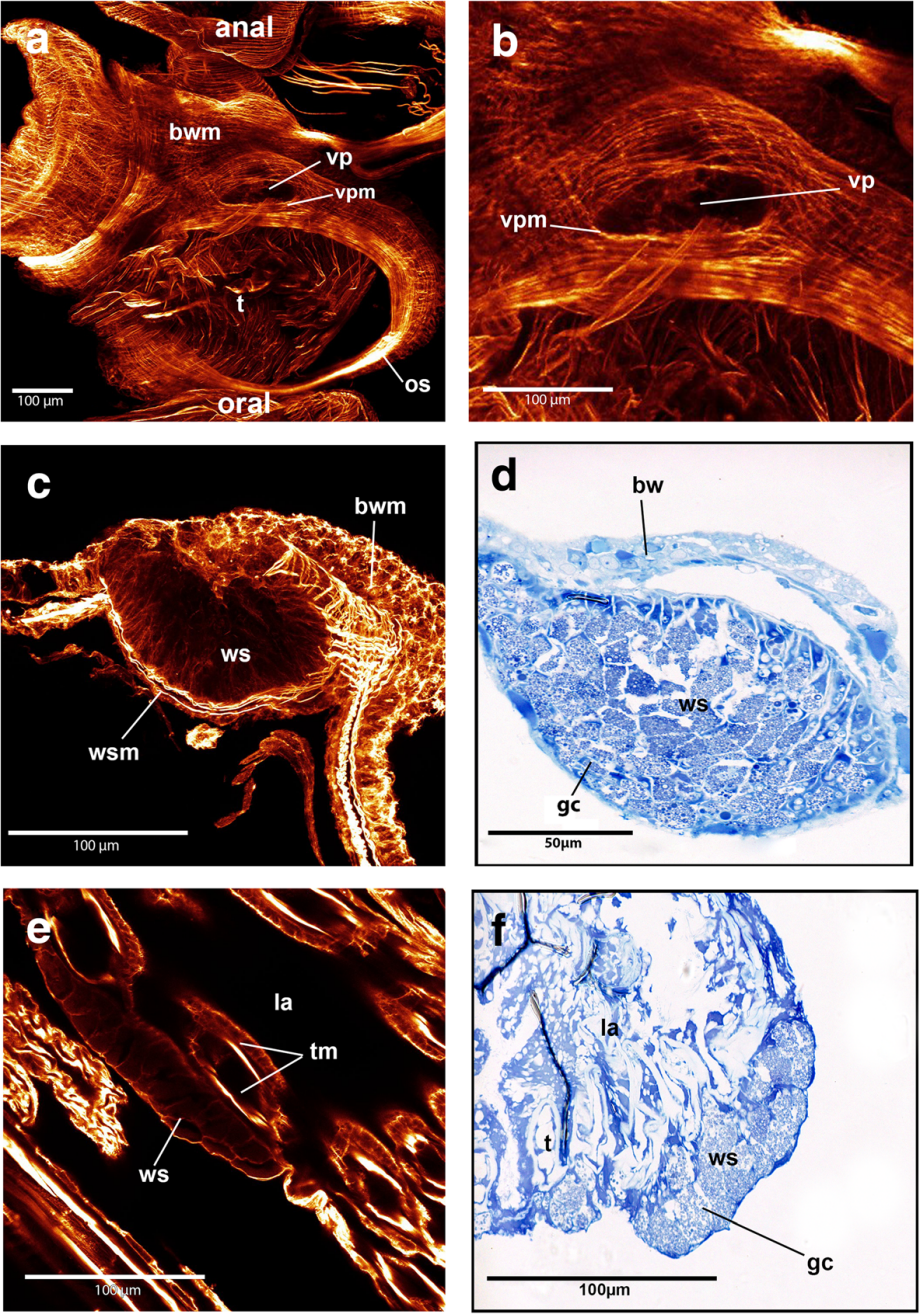
Maximum intensity projections of f-actin staining scans of the vestibular pores and white spots as well as semi-thin sections of white spots in *Pectinatella magnifica*. **a**: Overview of the vestibular pore which is situated at the anal side of the duplicature and surrounded by muscle fibers. **b**: Detail of vestibular pore associated with a white spot which is situated where the bulge is visible. **c**: Maximum intensity projection of the white spot which is situated at the anal side of the duplicature and is embedded in the body wall and surrounded by musculature. The glandular cells of the white spots are visible. **d**: Semi-thin section stained with toluidine blue of the white spot on the anal side of the duplicature. The glandular cells are visible. **e**: Maximum intensity projection of the white spot on the outside of the lophophoral arm. The glandular cells are visible and muscle fibers surrounding it are missing. (**f)**: Semi-thin section of the lophophoral white spot. The glandular cells are visible, which have the same appearance as the glandular cells of the white spot on the anal side of the duplicature. Abbreviations: bw – body wall, bwm – body wall musculature, gc - glandular cells, la - lophophoral arms, os - orifice sphincter, t - tentacles, tm – tentacle musculature, vp – vestibular pore, vpm - vestibular pore musculature, ws - white spot, wsm - white spot musculature

In several specimens of *Pectinatella magnifica* were complexes of glandular cells, called white spots, that could be identified at two sites: on the anal side of the duplicature, which is an invagination fold of the vestibular wall located around the orifice, and at the distal tip of the lophophoral arms (Fig. 9). White spots are visible on the surface of the colony as dots that generate a regular pattern in light microscopy images (Fig. 2c, d). A bulge in the body wall is visible on the anal side of the duplicature where a single white spot is visible (Fig. 9a, b). This white spot opens into a pore, which is surrounded by muscles forming a sphincter (Fig. 9a, b). This white spot is surrounded by muscles of the body wall. On the lophophoral arms the white spot is situated at the base of the tentacles. These white spots lack specific muscles and are not embedded into the body wall (Fig. 9e, f). All white spots are gland cell aggregations that contain small vesicles (Fig. 9d, f). In *C. mucedo* and *H. punctata* neither white spots nor a vestibular pore could be identified.

## Discussion

### Bodywall and tentacle sheath musculature in Phylactolaemata

Two configurations of the body wall musculature are present in phylactolaemate bryozoans: a regular mesh of two muscular layers (as in species of *Cristatella*, *Fredericella*, *Hyalinella*, *Lophopodella*, *Plumatella*) or three muscular layers (as in species of *Lophopus* and *Pectinatella*) in the body wall. The number of muscle layers of the body wall represents a difference in the myoanatomy in phylactolaemate bryozoans (Table 1.). The body wall musculature was first described as a mesh of transverse and longitudinal fibers [33]. Subsequent descriptions of the body wall musculature always showed two distinct layers in almost all investigated phylactolaemates (e.g. [34–36]). In general, four layers of the body wall can be distinguished: the epidermis, one outer layer of ring muscles, one inner layer of longitudinal muscles and the peritoneum. Interestingly, the two layers of muscles shows regional variations in thickness. In *Cristatella,* the more densily arranged muscles at the basal side form the creeping sole.

**Table 1:** Main differences in the myoanatomy of investigated Phylactolaemata. [26, 36, 37]. Two layers of the bodywall muscles are circular + longitudinal, while 3 layers also includes a diagonal one

	tentacle sheath muscles	bodywall muscles	epistome muscles	lophophoral arm muscles	ring canal muscles
*Pectinatella*	longitudinal + circular	3 layers	muscle fibers traversing the epistomal cavity	present + prominent	present
*Cristatella*	longitudinal + circular	2 layers	muscular basket	present + prominent	present
*Fredericella*	longitudinal + circular	2 layers	muscular basket	absent	not described
*Hyalinella*	mainly longitudinal	2 layers	muscular basket +muscle fibers traversing the epistomal cavity	present	present
*Plumatella*	mainly longitudinal	2 layers	muscular basket	present	not described
*Lophopodella*	not described	2 layers	muscle fibers traversing the epistomal cavity	not described	not described
*Lophopus*	longitudinal	3 layers	muscle fibers traversing the epistomal cavity	not described	not described

In Plumatellidae, including *H. punctata*, and in Fredericellidae, a two-layered regular grid of muscles has been described by fluorescence staining and CLSM analysis [26]. Two layers of muscles in the body wall were also recently confirmed in *Plumatella emarginata* by transmission electron microscopy [4]. Epidermal and peritoneal cells of the endocyst are myoepithelial cells that form the longitudinal and circular muscles [4]. The typical regular mesh of circular and longitudinal musculature exists also in *Cristatella mucedo*. In *Pectinatella magnifica* a third diagonal layer of muscles can be recognized in some parts of the body wall underneath the regular mesh of longitudinal and circular musculature. This third layer was already described for *P. magnifica* and *Lophopus crystallinus,* mainly in the basal, but also in lateral parts of the body wall [36]. The first two layers are organized as a regular, orthogonal mesh and the third layer is shifted by 45° relative to the other two layers [23, 34]. In contrast, a third layer appears to be lacking in *Lophopodella carteri* which, like *Lophopus crystallinus,* belongs to the Lophopodidae (Table 1) [37].

The tentacle sheath surrounds the tentacles when the animal is retracted [1]. There are differences between species of the Phylactolaemata with regards to the longitudinal and circular musculature of the tentacle sheath. (Table 1). Mostly longitudinal musculature was reported for the tentacle sheath of phylactolaemate freshwater bryozoans such as *Lophopus crystallinus* (e.g. [36]) or *Asajirella gelatinosa* [1]. *P. magnifica* and *C. mucedo* both possess longitudinal and circular muscles over the entire length of the tentacle sheath. Furthermore, both types of muscles are present in the tentacle sheath of *Fredericella sultana* [26]. Since Pectinatellidae and Cristatellidae are considered earlier lineages within Phylactolaemata [31] this could represent an ancestral feature of phylactolaemate bryozoans. This muscular arrangement reflects the condition found in the body wall and might be a plesiomorphic character shared by an ancestral or even pre-bryozoan form where the retraction process was not yet fully established and a continuous body wall covered the animal. In contrast, in several *Plumatella* species the ring musculature is restricted to the location where the tentacle sheath is attached to the lophophoral base and most of the circular musculature could have been secondarily reduced [26]. The current study reveals that another plumatellid, *Hyalinella punctata*, possess muscles in an identical position to species of *Plumatella*. Accordingly, three out of six phylactolaemate taxa possess circular musculature in the whole tentacle sheath. Furthermore, for all investigated gymnolaemates, only longitudinal musculature has been reported. More comparative data are necessary to assess whether ring musculature in the tentacle sheath evolved independently in these 3 phylactolaemate taxa or whether it is also present in other taxa. Longitudinal and circular musculature in the tentacle sheath is not correlated to the gelatinous colony type since it also occurs in the non-gelatinous *Fredericella sultana,* but is different to the gelatinous plumatellid *Hyalinella punctata* which lacks most of the circular muscles (see above)*.* Data from the earliest diverging clades, the Lophopodidae and Stephanellidae, would help to understand whether longitudinal and/or circular musculature is the ancestral state within Phylactolaemata.

### Musculature of the aperture in Phylactolaemata

In *Pectinatella magnifica*, *Cristatella mucedo* and *Hyalinella punctata* the prominent orifice sphincter is formed by a dense aggregation of circular muscles. The same situation is found in all other phylactolaemates such as *Fredericella sultana* and *Plumatella* sp. [26]. In general, the musculature associated with the aperture region always consists of several types of muscles in all phylactolaemates, the musculature of the vestibular wall, the vestibulum dilatators and the duplicature bands [25]. The vestibulum dilatators are always single smooth muscles [1]. The duplicature bands are composed of peritoneal tubes that contain longitudinal muscles; these prevent complete eversion of the animal [22]. The duplicature bands are attached at two different places in species of Phylactolaemata. In *Lophopus crystallinus* and *Lophopodella carteri* (Lophopodidae), the duplicature bands insert at the diaphragm, while in all other phylactolaemates [25], including *P. magnifica*, *C. mucedo*, and *H. punctata* (this study), the duplicature bands insert at the tentacle sheath. The situation of the musculature in the apertural region is consistent for all investigated phylactolaemate species containing musculature of the vestibular wall, duplicature bands and vestibulum dilatators.

### Retractor muscles in Phylactolaemata

Several combined sets of musculature and coordinate their contraction to effectively retract or protrude the polypide [3]. One set is the retractor muscles and their function is the retraction of the polypide into the cystid. They are the most prominent muscles of the animals [22]. The retractor muscles are paired and composed of longitudinal fibers. They originate from the body wall and insert at several lateral locations on the polypide. In *Lophopus crystallinus, Plumatella* sp. and *Cristatella mucedo* the retractor muscles were reported to insert at the lophophore and digestive tract, near the mouth opening as well as at the cardia and caecum. In *Pectinatella magnifica* the retractors insert at the caecum near the funiculus [36]. The recent study reveals that in *C. mucedo*, *P. magnifica* and *H. punctata* the retractor muscles consist of smooth fibers and are attached to the lophophoral base, ganglion, tentacle sheath and the gut, i.e. the pharynx, esophagus, cardia and caecum. The same situation was found for the retractor muscles in *Plumatella* sp.*.* In *F. sultana,* in contrast to other phylactolaemates, some distal parts of the fibres appear with regular striation [26]. Accordingly, the retractor muscles in phylactolaemates are very similar in morphology and function.

### White spots and vestibular pores in Phylactolaemata

The so-called vestibular pore was described in several phylactolaemate species including *Stolella evelinae, S. agilis, Hyalinella carvalhoi* [38, 39], *Plumatella fruticosa, P. repens, P. ermarginata* [40, 41] and *Lophopus crystallinus* [42]. In *Lophopus* the pore is surrounded by muscles, which indicates that the pore can be actively opened and closed [42]. This resembles the situation of the white spot located at the duplicature in *Pectinatella magnifica*. In all phylactolaemate species with a vestibular pore it is situated on the anal side of the duplicature. However, no vestibular pore could be found in *Cristatella mucedo* in this study.

The regular white structures of *P. magnifica* colonies were described as epidermal glands [23]. In *P. magnifica* and *Lophopodella carteri* these white spots or white bodies are filled with granular vesicles containing lipids and proteins. In *L. carteri* these white spots are located between two zooids embedded in the body wall [43]. White spots in *P. magnifica* are present at the distal tip of the lophophoral arms and on the anal side of the duplicature, where two of these glandular structures have been described [43]. Furthermore, only one white spot on the anal side of the duplicature could be identified in *P. magnifica* as well as in *Lophopus crystallinus*, in the latter called vestibular gland. It is not situated in the middle of the aperture but on the right or left side of the aperture [42]. This resembles the findings of the current study in *P. magnifica*. Noteworthy, several white spots are also present in on the oral side of *P. magnifica* larvae [43]. Since such glandular structures are only found in *P. magnifica,* which is commonly regarded as the sister group to *C. mucedo* [31], and two representatives of the Lophopodidae, these glandular structures have probably evolved independently. As in *Lophopus crystallinus*, the white spot on the anal side of the duplicature of *P. magnifica* is surrounded by muscles associated with a vestibular pore. Since vestibular pores were found in several phylactolaemate species they probably represent an ancestral character for this class [42].

### Musculature of the digestive tract and funiculus in Phylactolaemata

The typical u- shaped digestive tract possesses exclusively ring musculature in almost all phylactolaemates [36]. In *Cristatella mucedo*, *Hyalinella punctata* and *Pectinatella magnifica,* a typical arrangement of circular muscles with areas of denser aggregation at the pharynx and in the proximal part of the caecum is present. The same situation is present in the musculature of the gut of *Plumatella* sp. and *Fredericella sultana* [26]. Only in *Asajirella gelationsa* is longitudinal musculature present on the digestive tract [1]. Striation of the musculature is present in the foregut, especially the pharynx and caecum, of all investigated species [26]. Similar observations were also made in the present study. Since only circular musculature is present in almost all investigated phylactolaemates, it most likely represents the ancestral state for Phylactolaemata. Furthermore, the funiculus of all three species consists of a peritoneal cord with fine longitudinal muscles as previously demonstrated for *Plumatella* sp. and *Fredericella sultana* [26].

### Musculature of the lophophore in Phylactolaemata

Several sets of muscles are associated with the lophophore musculature of the epistome, tentacles, ring canal and lophophoral arms. In *Pectinatella magnifica*, *Cristatella mucedo* and *Hyalinella punctata* the situation in the epistomal musculature differs (Table 1). *C. mucedo* possesses a muscular basket in the epistomal wall whereas *P. magnifica* has prominent single fibers that traverse the coelomic cavity of the flap-like structure. This resembles the situation of *Lophopus crystallinus* [36] and *Lophopodella carteri* where the muscles traverse the epistomal cavity in anal to oral direction [37]. However, in a more recent study the presence of an epistome could not be confirmed in *Lophopus crystallinus* [4]. There is also a muscular basket that encompasses the epistome in *Plumatella* sp. and *Fredericella sultana* [26]. Interestingly, in *Hyalinella punctata* the musculature of the epistome possesses a unique condition among phylactolaemates: part of the muscles traverses the coelomic cavity and the other part is embedded in the epistomal wall orientated laterally. Accordingly, three situations of epistomal musculature can be found in Phylactolaemata (Table 1). It remains unclear if a muscular basket or fibers traversing the coelomic cavity of the epistome is the ancestral condition for phylactolaemates.

The tentacles of *P. magnifica*, *C. mucedo* and *H. punctata* possess two longitudinal muscle bands (this study), which is the same condition noted for *Fredericella sultana* and *Plumatella* sp. [26]. The muscles are generally smooth in most phylactolaemates, while in *C. mucedo* and *P. magnifica* they are striated. This may be synapomorphic for these two species considering their likely sister group relationship [31]. However, since most non-phylactolaemate bryozoans possess striated tentacle musculature (e.g. [1], Schwaha unpubl. Observations), it might also reflect the ancestral state with smooth musculature being the derived state. Detailed information about the muscular system in the lophophoral base and tentacle bases only exists for *Plumatella* sp. and *Fredericella sultana* [26]. Differences among investigated species is also present (Table 1): The large lophophoral arms possess a set of prominent longitudinal muscles in *P. magnifica* and *C. mucedo*. Here, the muscles branch off to the tentacles and terminate at the tentacle base [34]. This resembles the findings of the present study. In *C. mucedo* and *P. magnifica* two prominent muscle bundles are located on both sides of the ganglionic horns. These two bundles extend from the retractor muscles and run into the distal tip of the lophophoral arms. In *Plumatella* sp. the lophophoral arms along with their musculature appear less prominent and the extensions of the retractors into the lophophoral arms are lacking. As in *Plumatella* sp. the same situation could be confirmed for the lophophoral arms of the plumatellid *H. punctata. Fredericella sultana* has a circular lophophore and lacks lophophoral arm musculature altogether [26]. These extensions of the retractors into the lophophoral arms have never been described before and may represent a synapomorphy of *C. mucedo* and *P. magnifica.* More differences among phylactolaemate species in the myoanatomy exist in the tentacle bases. In *Plumatella* sp. the abfrontal bases are consistent over the whole length as in *C. mucedo, P. magnifica* and *H. punctata* and possess obliquely arranged muscles at the tentacle base. The muscle band is an inverted v-shape. In *P. magnifica* and *C. mucedo* two bundles of muscle fibers run along the lophophoral arms that branch off to the frontal rootlets of the tentacles situated on the margin of the lophophoral arms. Furthermore, in contrast to *Plumatella* sp. and *H. punctata, C. mucedo* and *P. magnifica* have one more rootlet at the base of the oral tentacles. In *C. mucedo* cross connections between the rootlets could be identified. It is possible that this is an apomorphy for *C. mucedo* since these cross connections are only described for this genus*.*

The ring canal is located at the base of the oral tentacles and is part of the lophophoral coelom. Up until now, ring muscles were unknown in phylactolaemates [26], but we have demonstrated their presence in *P. magnifica, C. mucedo* and *H. punctata* (Table 1). Its occurrence in three different families suggests that this musculature is also present in other phylactolaemate taxa. The gelatinous species are distinctly larger than the typical branching type of *Plumatella* and *Fredericella* species (also applies for the gelatinous plumatellid *Hyalinella punctata*) and possibly this musculature has been overlooked so far. Species of the early branching families Lophopodidae and Stephanellidae should be examined in the future to determine if lophophoral and ring canal muscles are present throughout the Phyloactolaemata.

### Comparative bryozoan myoanatomy

**Fig. 10:**
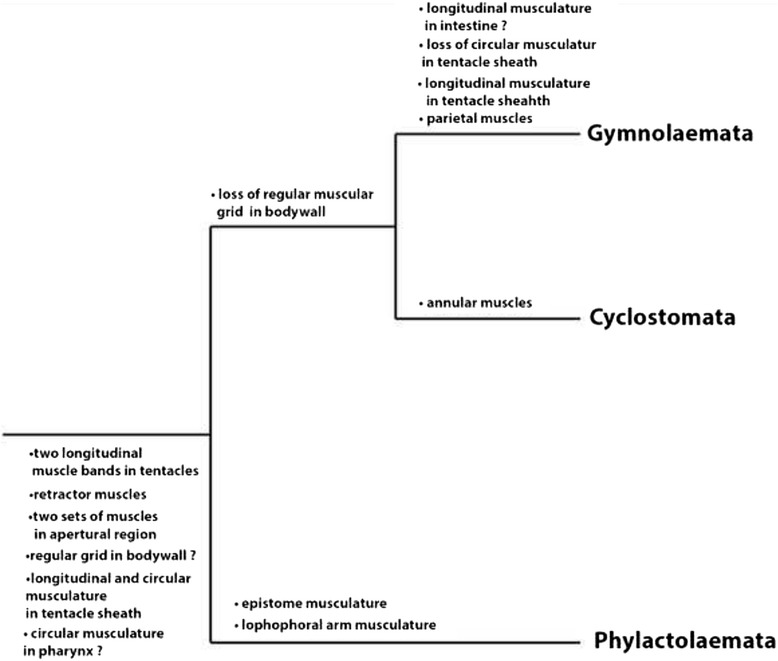
Summary of potential ancestral features of bryozoans and apomorphies for each bryozoan subtaxon in the muscular system

Recently, Phylactolaemata was confirmed as the earliest branch within bryozoans and thus represents an important taxon for the reconstruction of the ground pattern of Bryozoa [21]. Data concerning the myoanatomy is almost completely lacking in the Cyclostomata and available to a greater extent for Phylactolaemata and Gymnolaemata. Some ancestral features, but also apomorphies of each bryozoan class-level taxon, can be identified (Fig.10). First of all, phylactolaemates possess a regular orthogonal grid of body wall musculature in contrast to members of the other two taxa, the Stenolaemata and Gymnolaemata [1]. Despite the lack of typical body wall musculature in Stenolaemata and Gymnolaemata, some muscle groups in these two taxa are derived from the body wall musculature of phylactolaemates. In Cyclostomata so called annular muscles exist in the wall of the membranous sac and are formed by thin fibers that are located between the outer basal membrane and the inner peritoneal layer. In the Gymnolaemata the parietal muscles which are located in series at the lateral position on each side have also probably evolved from body wall musculature [44].

In *Pectinatella magnifica*, *Cristatella mucedo* and *Fredericella sultana* longitudinal and circular musculature could be identified in the whole tentacle sheath. Considering that the body wall musculature is arranged in the same way as in the tentacle sheath in these three species, this probably represents the ancestral condition for bryozoans. The last common ancestor of all recent bryozoans possibly had no or restricted ability of retracting the whole zooid into the cystid and the body wall was continuous without any duplicature which provides a connection between body wall and tentacle sheath [44]. This also indicates that the arrangement of the tentacle sheath muscles with longitudinal and circular fibers represents the ancestral condition [44]. Since the tentacle sheath has only longitudinal musculature in the later branching Gymnolaemata [25], this condition could be more derived and the circular musculature secondarily reduced.

A second distinct difference between the major taxa can be found in the digestive tract where in phylactolaemates exclusively dense ring musculature can be found in the whole digestive tract which, to a large extent, is striated. Data from the Cyclostomata is missing. Gymnolaemates have a promiment muscular foregut consisting mainly of striated circular muscles with few smooth longitudinal fibers [1], whereas the remaining gut possesses a rather loose, thin basket of smooth muscles [25, 45].

Concerning the musculature of the pharynx, phylactolaemates have mainly striated circular musculature with the exception of *Asajirella gelatinosa* where additional longitudinal muscles are present [1]. In Cyclostomata mainly striated and circular pharyngeal musculature with a few longitudinal fibers exists [46, 47]. In gymnolaemates also striated pharyngeal ring musculature is typical [25, 45, 48]. Since in almost all investigated bryozoans striated circular musculature in the pharynx is present it probably represents the ancestral condition.

All bryozoans possess retractor muscles that consist of longitudinal muscles originating from the body wall and inserting at the polypide [1]. Most phylactolaemates have exclusively smooth muscles in the retractors, but in *Fredericella sultana*, some fibers appear striated at their distal tips [26]. However, in investigated cyclostomes [46] and some cheilostome gymnolaemates [35] they appear entirely striated, but also smooth retractor musculature exists in some cheilostome species [49] as well as in ctenostomes species [25, 45]. Most reports of striated retractor muscles come from older observations that relied on conventional microscopy and histology, but more recent analyses using CLSM have shown that most retractors have smooth patterns.

A set of apertural musculature is always present in all the three bryozoan class-level taxa. Several sets of muscles can be homologized based on position and structure. All three groups possess an invaginated fold which forms the vestibular wall that is separated from the tentacle sheath by a diaphragmatic sphincter [25]. Phylactolaemate species possess vestibulum dilatators which are individual fibres that traverse the coelom between body wall and vestibular wall, and duplicature bands which are peritoneal bands with several smooth muscles located between body wall and diaphragmatic sphincter (Lophopodidae) or tentacle sheath (rest of Phylactolaemata) [25]. Cyclostomes possess a unique condition of the peritoneum. The peritoneal layer forms the membranous sac which surrounds the polypide [50]. In several cyclostomes the attachment organ fixes the sac and the polypide to the calcified walls and is topologically and structurally similar to the peritoneal duplicature bands. Several muscles between the body wall and the diaphragm form the vestibulum dilatators in cyclostomes. The parieto-vaginal bands of gymnolaemates are homologous to the duplicature bands of phylactolaemates. Furthermore, the prominent vestibular muscles such as the operculum occlusor are homologous to the vestibulum dilatators of phylactolaemates [25].

Phylactolaemata is the only bryozoan class-level taxon with a horseshoe-shaped lophophore and an epistome, the other two classes typically have a circular lophophore and lack an epistome [1] (Mukai et al. 1997). Accordingly, the musculature of the lophophoral arms and epistome is present only in Phylactolaemata. Similar to the phylactolaemates, cyclostomes and gymnolaemates also possess two longitudinal muscle bands in the tentacles which are located at the frontal and abfrontal side. Previous investigations found them to be smooth muscles in phylactolaemates [4, 26] whereas the current study shows striated patterns in *C. mucedo* and *P. magnifica*. In cyclostomes [46, 51] and in ctenostomes [25, 45, 52] they are likewise reported to be striated.

### Comparison of the myoanatomy of the three lophophorate clades Bryozoa, Phoronida and Brachiopoda

Members of the three phyla share some distinct morphological features such as the lophophore with an epistome and tentacles and were traditionally united as the Lophophorata [6]. The epistome of phylactolaemate bryozoans always possesses an extension of the visceral coelomic cavity [4]. However, the presence of an epistomal coelom in phoronids and brachiopods is controversial [53–55]. A recent study showed that the epistome of phoronids [55, 56] and brachiopods [57] possesses a coelomic compartement but in the past the epistome of brachiopods [53] and phoronids [54] was described as lacking a coelomic cavity. Nevertheless, in all three groups musculature in the epistome can be found (e.g. [26, 53, 54, 57, 58]). In phoronids the epistome is either filled with myoepithelial cells positioned in the lateral walls of the epistome [54] or the myoepithelial cells form a basket including fibres traversing the cavity as observed in bryozoans [55]. In brachiopods muscle cells contain myofilaments that are orientated along the epistome [57]. Most descriptions for non-bryozoan lophophorates mostly argue for a muscular basket lining in the form of myoepithelial cells that corresponds to the situation found in *C. mucedo*, partially *H. punctata* (this study) as well as *Plumatella* sp. and *Fredericella sultana* [26]. However, especially since phoronids sometimes show a mixture of both types of fibres, it appears that a mixed set would be the ancestral state for lophophorates.

Regular body wall musculature can be found in bryozoans (e.g. [1]) and phoronids [55] but is missing in brachiopods [59]. Phoronids and phylactolaemate bryozoans both possess an outer circular and an inner longitudinal muscle layer in the body wall, of which the longitudinal one is more prominent in phoronids [60]. Recently, a third layer of diagonal muscle, also found in some phylactolaemate species (Table 1), was also detected in phoronids [61]. In brachiopods the mantle lacks a regular mesh of musculature and possesses specialized musculature such as the mantle margin musculature as an adaption to the two shells [59].

Data on lophophoral musculature in brachiopods and phoronids is sparse and mostly restricted to studies on the tentacle musculature. In all three groups longitudinal muscle bands are situated on the frontal and abfrontal side. In brachiopods [62, 63] and phoronids [64] myoepithelial cells are also concentrated on the frontal and abfrontal side end extend along the tentacles as in bryozoans [26].

Since different phylogenetic scenarios are proposed for the lophophorate taxa (e.g. [12, 14]) it remains questionable whether these similarities in the muscular system support the Lophophorata-concept or evolved independently. Recent morphological studies show similarities in the nervous system of the three lophophorate groups in support for a monophyletic clade [16, 17]. For example, the cerebral ganglion of bryozoans is considered to be homologous to the dorsal ganglion of phoronids and to the brachial nerve of brachiopods [16]. Furthermore, lophophore innervation such as the tentacle innervation and the main nerves are similar among bryozoans, phoronids and brachiopods which, possibly along with the entire lophophore, represent homologous structures [17]. However, as mentioned above, only two recent phylogenies currently show only support for a monophyletic Lophophorata (e.g. [14, 15]).

## Conclusions

This study provides data on the myoanatomy of three gelatinous phylactolaemate representatives in order to gain more information on the muscular ground pattern of Phylactolaemata and bryozoans in general. Furthermore, it represents the first description of ring canal musculature in species of the Phylactolaemata. Synapomorphies for *Cristatella mucedo* and *Pectinatella magnifica,* which have recently been claimed to be sister groups, could be identified; i.e. the extensions of the retractor muscles in the lophophoral arms and striated tentacle musculature. For *Hyalinella punctata* the unique situation in the epistome could represent an apomorphic character for this species or the whole genus. We show that the muscular system in all phylactolaemates is very similar, differences exist mainly in the body wall, epistome, lophophoral base and ring canal (Table. 1). For the three bryozoan class-level taxa several potential ancestral features exist concerning their myoanatomy: circular and striated musculature in the pharynx, two muscle bands in the tentacles, circular and longitudinal musculature in the tentacle sheath, a pair of retractor muscles, the musculature of the apertural region and a regular grid of body wall musculature (Fig. 10). Apomorphic muscular features are identifiable for each bryozoan taxon (Fig. 10). The regular grid in the body wall exists in the early diverging phylactolaemates and lacks in cyclostomes and gymnolaemates. In the lophophore several muscles are restricted to Phylactolaemata, for example the epistome musculature and musculature of the lophophoral arms. For Cyclostomata the annular ring muscles of the membranous sac represent an autapomorphy. Another difference between the three taxa is in the digestive tract, where phylactolaemates possess exclusively ring musculature in the whole gut and in gymnolaemates the intestine has only smooth, longitudinal musculature, which represents a potential apomorphy for Gymnolaemata. In phylactolaemates and cyclostomes the tentacle sheath contains longitudinal and circular muscles, whereas the tentacle sheath of gymnolaemates contains solely longitudinal muscles.

Additional data on the muscular system from the earliest branching phylactolaemate families, the Stephanellidae and Lophopodidae and also from representatives of the other two bryozoan subtaxa, especially from the cyclostomes, are needed for a solid assessment of the muscular ground plan of this group and to enable a more profound comparison of character evolution of the three bryozoan subtaxa.

## Additional file

Additional file 1: Fig S1. Maximum intensity projections of f-actin staining scans of *Hyalinella punctata*. A: The base of the frontal oral tentacle muscle band consists of two rootlets which do not originate from the pharyngeal epithelium but from a ring muscle surrounding the pharynx. B: The ring canal which is located at the base of the oral tentacles possesses own musculature at the base. Sometimes a single, additional fiber is traversing the ring canal medially in this species. C: Lateral view of one zooid showing frontal tentacle bands with one rootlet at the base. Delicate muscles are visible at the base of the lophophoral arm between pharynx and intestine. D: View of the lophophoral arms from the anal side. Muscle fibers with weak signal are visible at the base of the lophophoral arms. Abbreviations: arc - additional ring canal muscle, bwm – body wall musculature, cae – caecum, int – intestine, ftbm – frontal tentacle base muscle, ftmb – frontal tentacle muscle band, lam – lophophoral arm musculature, mphx – muscle surrounding the pharynx, phx – pharynx, rc - ring canal, rcm - ring canal musculature, rm. – retractor muscle, t – tentacle, ts – tentacle sheath. (TIFF 13265 kb)

## References

[1] Mukai H, Terakado K, Reed CG, Harrison FW, Woollacott RM (1997). Bryozoa. Microscopic anatomy of invertebrates.

[2] Silen L (1944). On the division and movements of the alimentary canal of the Bryozoa. Ark Zool.

[3] Wood TS, Robinson RA (1983). General features of the class Phylactolaemata. Treatise on invertebrate Palaeontology part G: Bryozoa (revised).

[4] Gruhl A, Wegener I, Bartolomaeus T (2009). Ultrastructure of the body cavities in Phylactolaemata (Bryozoa). J Morphol.

[5] Wood TS: Phyla Ectoprocta and Entoprocta (bryozoans). In: Ecology and general biology, Vol I: thorp and Covich's freshwater invertebrates, 4th Edition. Edited by Thorp JH, Rogers DC. London: Academic Press; 2014: 327-345.

[6] Emig CC (1984). On the origin of the Lophophorata. Zeitschrift für zoologische Systematik und Evolutionsforschung.

[7] Halanych KM, Bacheller JD, Aguinaldo AMA, Liva SM, Hillis DM, Lake JA (1995). Evidence from 18S ribosomal DNA that lophophorates are protostome animals. Science.

[8] Philippe H, Lartillot N, Brinkmann H (2005). Multigene analyses of Bilaterian animals corroborate the Monophyly of Ecdysozoa, Lophotrochozoa, and Protostomia. Mol Biol Evol.

[9] Brusca RC, Brusca GJ. Invertebrates. Sunderland, MA: Sinauer Press; 2003.

[10] Hausdorf B, Helmkampf M, Meyer A, Witek A, Herlyn H, Bruchhaus I, Hankeln T, Struck TH, Lieb B (2007). Spiralian phylogenomics supports the resurrection of Bryozoa comprising Ectoprocta and Entoprocta. Mol Biol Evol.

[11] Helmkampf M, Bruchhaus I, Hausdorf B (2008). Multigene analysis of lophophorate and chaetognath phylogenetic relationships. Mol Phylogenet Evol.

[12] Mallatt J, Craig CW, Yoder MJ (2012). Nearly complete rRNA genes from 371 Animalia: updated structure-based alignment and detailed phylogenetic analysis. Mol Phylogenet Evol.

[13] Helmkampf M, Bruchhaus I, Hausdorf B (2008). Phylogenomic analyses of lophophorates (brachiopods, phoronids and bryozoans) confirm the Lophotrochozoa concept. Proc R Society B-Biol Sci.

[14] Nesnidal MP, Helmkampf M, Meyer A, Witek A, Bruchhaus I, Ebersberger I, Hankeln T, Lieb B, Struck TH, Hausdorf B. New phylogenomic data support the monophyly of Lophophorata and an Ectoproct-Phoronid clade and indicate that Polyzoa and Kryptrochozoa are caused by systematic bias. BMC Evol Biol. 2013;13:253. 10.1186/1471-2148-13-253 PMC4225663 24238092

[15] Nesnidal MP, Helmkampf M, Bruchhaus I, Ebersberger I, Hausdorf B, Wagele JW, Berlin BT (2014). Lophophorata monophyletic - after all. Deep metazoan phylogeny: the backbone of the tree of life: new insights from analyses of molecules, morphology, and theory of data analysis.

[16] Temereva EN, Tsitrin EB. Modern data on the Innervation of the Lophophore in Lingula anatina (Brachiopoda) support the Monophyly of the Lophophorates. PLoS One. 2015;10:e0123040. 10.1371/journal.pone.0123040 PMC4406759 25901745

[17] Temereva EN, Kosevich IA. The nervous system of the lophophore in the ctenostome *Amathia gracilis* provides insight into the morphology of ancestral ectoprocts and the monophyly of the lophophorates. BMC Evol Biol. 2016;16:181. 10.1186/s12862-016-0744-7 PMC5012098 27600336

[18] Temereva EN (2017). Morphology evidences the lophophorates monophyly: brief review of studies on the lophophore innervation. Invert Zool.

[19] Mundy SP, Taylor PD, Thorpe JP, Larwood GP, Nielsen C (1981). A reinterpretation of phylactolaemate phylogeny. Recent and fossil Bryozoa.

[20] Fuchs J, Obst M, Sundberg P (2009). The first comprehensive molecular phylogeny of Bryozoa (Ectoprocta) based on combined analyses of nuclear and mitochondrial genes. Mol Phylogenet Evol.

[21] Waeschenbach A, Taylor PD, Littlewood DTJ (2012). A molecular phylogeny of bryozoans. Mol Phylogenet Evol.

[22] Hancock A (1850). On the anatomy of the fresh-water Bryozoa, with descriptions of new species. Ann and Mag of Nat Hist ser 2.

[23] Kraepelin K: Die deutschen Süßwasser-bryozoen. 1. Anatomisch-systematischer Teil. Abhandlungen aus dem Gebiete der Naturwissenschaften, hrsg vom Naturwissenschaftlicher Verein in Hamburg 1887, 10: 168p.

[24] Schwaha T, Handschuh S, Redl E, Walzl M (2011). Organogenesis in the budding process of the freshwater bryozoan *Cristatella mucedo* Cuvier 1789 (Bryozoa, Phylactolaemata). J Morphol.

[25] Schwaha T, Wood TS, Wanninger A (2011). Myoanatomy and serotonergic nervous system of the ctenostome *Hislopia malayensis*: evolutionary trends in bodyplan patterning of Ectoprocta. Front Zool.

[26] Schwaha T, Wanninger A (2012). Myoanatomy and serotonergic nervous system of plumatellid and fredericellid Phylactolaemata (Lophotrochozoa, Ectoprocta). J Morphol.

[27] Shunkina KV, Zaytseva OV, Starunov VV, Ostrovsky AN. Comparative morphology of the nervous system in three phylactolaemate bryozoans. Front Zool. 2015;12:28. 10.1186/s12983-015-0112-2 PMC4603689 26464575

[28] Weber AV, Wanninger A, Schwaha TF. The nervous system of *Paludicella articulata* - first evidence of a neuroepithelium in a ctenostome ectoproct. Front Zool. 2014;11:89. 10.1186/s12983-014-0089-2 PMC4269932 25525454

[29] Gruhl A (2010). Neuromuscular system of the larva of *Fredericella sultana* (Bryozoa: Phylactolaemata). Zool Anz - J Comp Zool.

[30] Schwaha T, Handschuh S, Redl E, Wanninger A (2015). Insights into the organization of plumatellid 'larvae' (Lophotrochozoa, Bryozoa) by means of 3D imaging and confocal microscopy. J Morphol.

[31] Hirose M, Dick MH, Mawatari SF: Molecular phylogenetic analysis of phylactolaemate bryozoans based on mitochondrial gene sequences. In: Proceedings of the 14th International Bryozoology Association Conference, Boone, North Carolina, July 1–8, 2007, Virginia Museum of Natural History Special Publication No 15. Edited by Hageman SJ, Key MMJ, Winston JE. Martinsville, Virginia: Virginia Museum of Natural History; 2008: 65-74.

[32] Schindelin J, Arganda-Carreras I, Frise E, Kaynig V, Longair M, Pietzsch T, Preibisch S, Rueden C, Saalfeld S, Schmid B (2012). Fiji: an open-source platform for biological-image analysis. Nat Methods.

[33] Allman GJ (1856). A monograph of the fresh-water Polyzoa. Ray Soc Lond.

[34] Hyatt A (1865). Observations on polyzoan order Phylactolaemata. Proc Essex Inst.

[35] Nitsche H (1871). Beiträge zur Kenntnis der Bryozoen 3. Über die Anatomie und Entwicklungsgeschichte von Flustra membranacea 4. Über die Morphologie der Bryozoen. Z Wiss Zool.

[36] Marcus E (1934). Über *Lophopus crystallinus* (PALL.). Zool Jb Anat.

[37] Rogick MD (1937). Studies on fresh-water Bryozoa VI. The finer anatomy of Lophopodella Carteri. Trans Am Microsc Soc.

[38] Marcus E (1941). Sobre Bryozoa do Brasil. I. Boletim da Faculdade de filosofia, ciéncias e letras, Universidade di Sao Paolo. Zoologia.

[39] Marcus E (1942). Sobre Bryozoa do Brasil. II. Boletim da Faculdade de filosofia, ciéncias e letras, Universidade di Sao Paolo. Zoologia.

[40] Wiebach F (1952). Über den Ausstoß von Flottoblasten bei *Plumatella fructicosa*. ZoolAnz.

[41] Wiebach F (1953). Über den Ausstoss von Flottoblasten bei Plumatellen. Zool Anz.

[42] Gruhl A, Ernst A, Schäfer P, Scholz J (2013). Occurrence and identity of "white spots" in Phylactolaemata. Bryozoan studies 2010.

[43] Mukai H, Oda S (1980). Histological and histochemical studies on the epidermal system of higher phylactolaemate bryozoans. Annot Zool Jap.

[44] Jebram D (1986). Arguments concering the basal evolution of the Bryozoa. Z zool Syst Evoltut-forsch.

[45] Grischenko AV, Chernyshev AV (2015). *Triticella minini* - a new ctenostome bryozoan from the abyssal plain adjacent to the Kuril-Kamchatka trench. Deep-Sea Res Part Ii-Top Stud Oceanography.

[46] Borg F (1926). Studies on recent cyclostomatous Bryozoa. Zool Bidr Uppsala.

[47] Nielsen C (2013). The triradiate sucking pharynx in animal phylogeny. Invertebr Biol.

[48] Bullivant JS, Bils RF (1968). The pharyngeal cells of *Zoobotryon verticillatum* (delle Chiaje), a gymnolaemate bryozoan. NZ J Mar Freshw Res.

[49] Calvet L: Contribution à l'histoire naturelle des Bryozaires Ectoproctes marins. Travaux de l'institut de zoologie de l'Université de Montpellier et de la station zoologique de Cette NS 1900, 8: 1-488.

[50] Nielsen C, Pedersen KJ (1979). Cystid structure and protrusion of the polypide in *Crisia* (Bryozoa, Cyclostomata). Acta Zool.

[51] Nielsen C, Riisgard HU (1998). Tentacle structure and filter-feeding in *Crisia eburnea* and other cyclostomatous bryozoans, with a review of upstream-collecting mechanisms. Mar Ecol Prog Ser.

[52] Smith LW, Larwood GP (1973). Ultrastructure of the tentacles of *Flustrellidra hispida* (Fabricius). Living and fossil Bryozoa.

[53] Luter C (1996). The median tentacle of the larva of *Lingula anatina* (Brachiopoda) from Queensland, Australia. Aust J Zool.

[54] Gruhl A, Grobe P, Bartolomaeus T (2005). Fine structure of the epistome in *Phoronis ovalis*: significance for the coelomic organization in Phoronida. Invertebr Biol.

[55] Temereva EN, Malakhov VV (2011). Organization of the epistome in *Phoronopsis harmeri* (Phoronida) and consideration of the coelomic organization in Phoronida. Zoomorphology.

[56] Temereva EN. Organization of the coelomic system in *Phoronis australis* (Lophotrochozoa: Phoronida) and consideration of the coelom in the lophophorates. J Zool. 2015;296:79–94.

[57] Temereva EN, Gebruk AA, Malakhov VV (2015). Demonstration of the preoral coelom in the brachiopod *Lingula anatina* with consideration of its phylogenetic significance. Zool Anz.

[58] Pross A (1974). Untersuchungen über die Muskulatur des Epistoms der phoroniden *Phoronis ijimai* Oka (Phoronidea). Zool Jb Anat.

[59] Altenburger A, Wanninger A. Comparative larval myogenesis and adult myoanatomy of the rhynchonelliform (articulate) brachiopods Argyrotheca Cordata, A*. cistellula*, and *Terebratalia transversa*. Front Zool. 2009;6:3. 10.1186/1742-9994-6-3 PMC2645390 19192287

[60] Herrmann K, Harrison FW, Woollacott RM (1997). Phoronida. Microscopic anatomy of invertebrates.

[61] Chernyshev A, Temereva E (2010). First report of diagonal musculature in phoronids (Lophophorata: Phoronida). Dokl Biol Sci.

[62] Reed CG, Cloney RA (1977). Brachiopod tentacles: ultrastructure and functional significance of the connective tissue and myoepithelial cells in *Terebratalia*. Cell Tissue Res.

[63] Temereva EN (2017). Ultrastructure of the coelom in the brachiopod *Lingula anatina*. J Morphol.

[64] Pardos F, Roldan C, Benito J, Aguirre A, Fernandez I (1993). Ultrastructure of the Lophophoral tentacles in the genus *Phoronis* (Phoronida, Lophophorata). Canadian Journal of Zoology-Revue Canadienne De Zoologie.

