# Supplementary figures and images for: Reconstructing the muscular ground pattern of phylactolaemate bryozoans: first data from gelatinous representatives
Source: BMC Evol Biol. 2017 Nov 7;17:225. doi: 10.1186/s12862-017-1068-y (PMC5688826; doi:10.1186/s12862-017-1068-y)

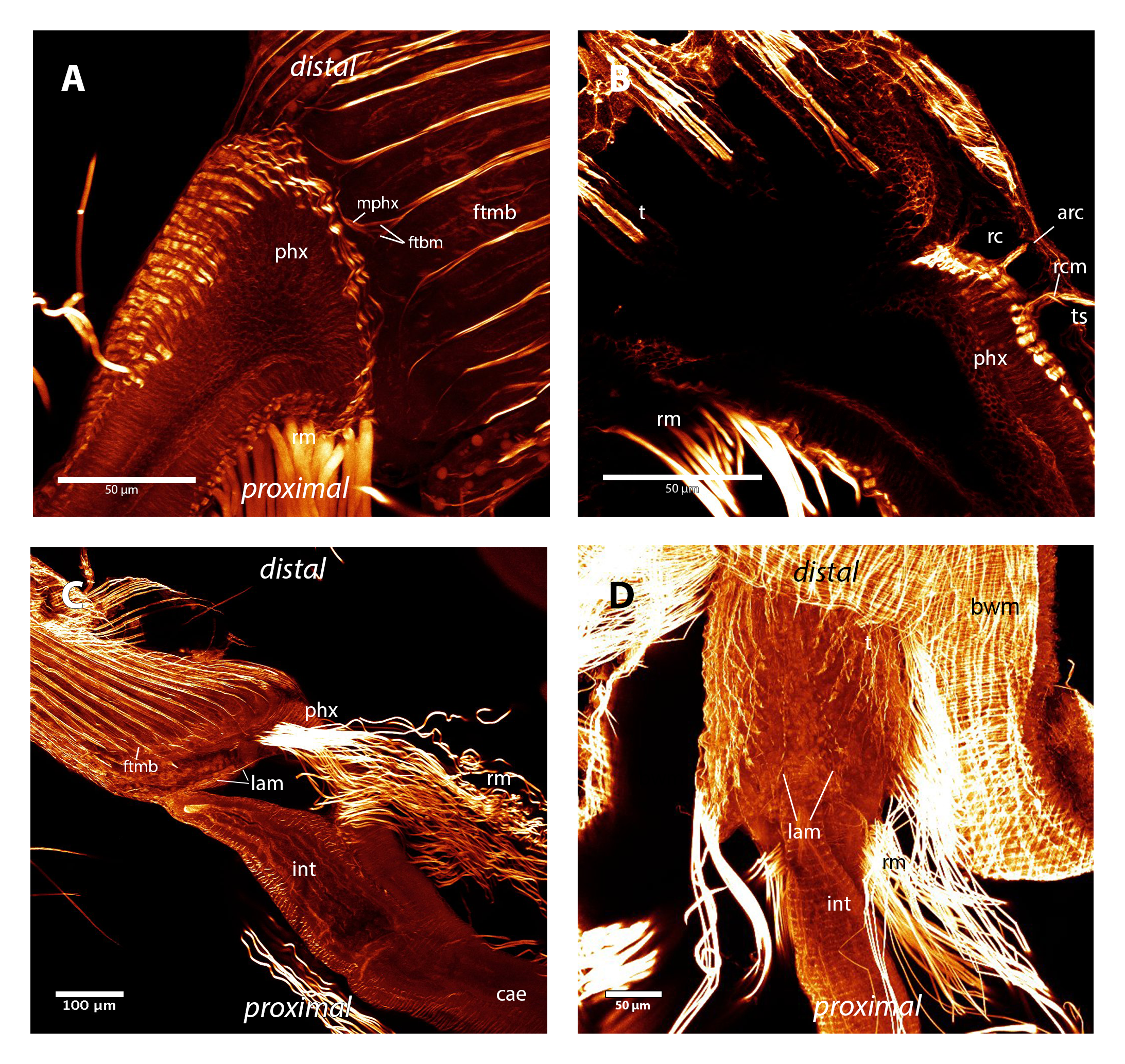

Supplement: Additional file 1: Fig S1. — Maximum intensity projections of f-actin staining scans of Hyalinella punctata. A: The base of the frontal oral tentacle muscle band consists of two rootlets which do not originate from the pharyngeal epithelium but from a ring muscle surrounding the pharynx. B: The ring canal which is located at the base of the oral tentacles possesses own musculature at the base. Sometimes a single, additional fiber is traversing the ring canal medially in this species. C: Lateral view of one zooid showing frontal tentacle bands with one rootlet at the base. Delicate muscles are visible at the base of the lophophoral arm between pharynx and intestine. D: View of the lophophoral arms from the anal side. Muscle fibers with weak signal are visible at the base of the lophophoral arms. Abbreviations: arc - additional ring canal muscle, bwm – body wall musculature, cae – caecum, int – intestine, ftbm – frontal tentacle base muscle, ftmb – frontal tentacle muscle band, lam – lophophoral arm musculature, mphx – muscle surrounding the pharynx, phx – pharynx, rc - ring canal, rcm - ring canal musculature, rm. – retractor muscle, t – tentacle, ts – tentacle sheath. (TIFF 13265 kb) [file 12862_2017_1068_MOESM1_ESM.tif]
